# Electronic and structural data of 4’-substituted bis(2,2’;6’2’’-terpyridine)manganese in *mono*-, *bis*-, *tris*- and *tetra*-cationic states from DFT calculations

**DOI:** 10.1016/j.dib.2022.108221

**Published:** 2022-04-30

**Authors:** Jeanet Conradie

**Affiliations:** a Department of Chemistry, University of the Free State, P.O. Box 339, Bloemfontein 9300, South Africa; b Department of Chemistry, UiT - The Arctic University of Norway, Tromsø N-9037, Norway

**Keywords:** Bis(terpyridine)manganese, Broken symmetry, Jahn-Teller, DFT

## Abstract

This data article provides density functional theory calculated structural (bond lengths and angles, coordinates of optimized geometries) and electronic (Mulliken spin population and character of frontier molecular orbitals) data of a series of 4’-substituted bis(2,2’;6’2’’-terpyridine)manganese complexes in four different oxidation states. The *bis*-cationic (*n* = 2) [Mn(tpy)_2_]^2+^ complexes are experimentally well known (Sjödin et al., 2008), while little or none experimental structural data of the *tetra*-cationic (*n* = 4, Romain et al., 2009, 2009), *tris*-cationic (*n* = 3, Romain et al., 2009) and *mono*-cationic (*n* = 1, Wang et al., 2014) [Mn(tpy)_2_]^n+^ complexes are available. For more insight into the provided data, see related research article “Redox chemistry of bis(terpyridine)manganese(II) complexes – a molecular view” (Conradie, 2022).

## Specifications Table

**Table:** 

Subject	Chemistry
Specific subject area	Physical and Theoretical Chemistry
Type of data	Table, Image, Graph, Figure
How the data were acquired	Geometry optimizations and electronic structure calculations were done using the quantum computational chemistry program Gaussian 16, Revision B.01.
Data format	Raw, Analyzed, Filtered
Description of data collection	DFT calculations were performed using the resources of the High-Performance Computing facility of the UFS, the CHPC of South Africa and the Norwegian Supercomputing UNINETT Sigma2 facility FRAM.
Data source location	University of the Free State, Bloemfontein, South Africa
Data accessibility	Output files of the DFT calculations, containing information on the optimized geometry, Mulliken spin populations and five frontier molecular orbitals (including HOMO and LUMO) are uploaded as four sets to the figshare data repository at the links:https://ufs.figshare.com/articles/dataset/Reduced_bis_terpyridine_manganese_II_complexes/19575667https://ufs.figshare.com/articles/dataset/Bis_terpyridine_manganese_II_complexes/19575619https://ufs.figshare.com/articles/dataset/Bis_terpyridine_manganese_III_complexes/19575631https://ufs.figshare.com/articles/dataset/Bis_terpyridine_manganese_IV_complexes/19575637
Related research article	J. Conradie, Redox chemistry of bis(terpyridine)manganese(II) complexes – A molecular view, J. Electroanal. Chem. 913 (2022) 116,272.

## Value of the Data

• The data reported in this work will save computational time to calculate the structural and electronic structure of the *tetra*-cationic (*n* = 4), *tris*-cationic (*n* = 3), *bis*-cationic (*n* = 2) and *mono*-cationic (*n* = 1) [Mn(tpy)_2_]^n+^ complexes. The optimization of these geometries easy led to higher energy local minima structures when starting with a different input geometry. Some jobs ran more than 2 weeks on 2 nodes with 16 processors each before they converged. Little or no experimental structural data of these complexes are available [1], [2], [3], [4].
• This data gives experimental chemists insight into the expected stability and reactivity of *mono*-, *bis*-, *tris*- and *tetra*-cationic states of [Mn(tpy)_2_]^n+^ complexes, that go through different oxidation states during catalytic cycles and electrochemical oxidation and reduction processes [5]. Transition metal-terpyridine complexes exhibit anti-microbial potential, are used in biomedical applications, and have unique optical, photo-luminescence-, catalytic-, photovoltaic-, sensitizers and sensor properties [6], [7], [8], [9].
• This data provides the geometry of the basic structure of the ground states of *mono*-, *bis*-, *tris*- and *tetra*-cationic states of [Mn(tpy)_2_]^n+^ complexes, that include broken symmetry, constrained octahedral and compression Jahn-Teller geometries. The data can be used for the determination of the geometrical and electronic structures of related [Mn(tpy)_2_]^n+^ complexes, containing other tpy ligands.

## 1 Data Description

**Scheme 1:**
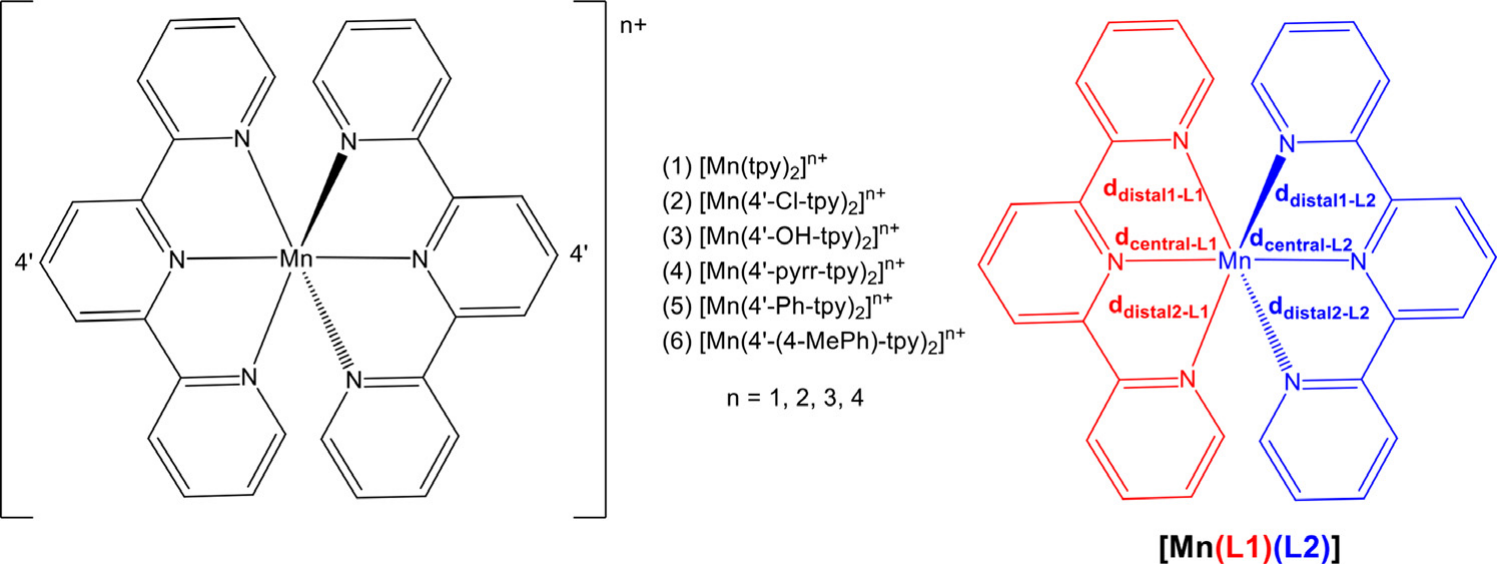
Bis(2,2’;6’2’’-terpyridine)manganese (1) and the series of 4’-substituted bis(2,2’;6’2’’-terpyridine)manganese complexes (2–6) of this study. Numbering of terpyridine ligands and notation used to distinguish between the Mn-N bonds, indicated.

The data obtained from computing the ground state geometries of *mono*-cationic (*n* = 1, high spin *S* = 2, S_Mn_ = 5/2), *bis*-cationic (*n* = 2, high spin *S* = 5/2), *tris*-cationic (*n* = 3, high spin *S* = 2) and *tetra*-cationic (*n* = 4, intermediate spin *S* = 3/2) states of [Mn(tpy)_2_]^n+^ complexes, containing different 4’-substituted 2,2’;6’2’’-terpyridine ligands, see Scheme 1, is reported in this work.

**Fig. 1:**
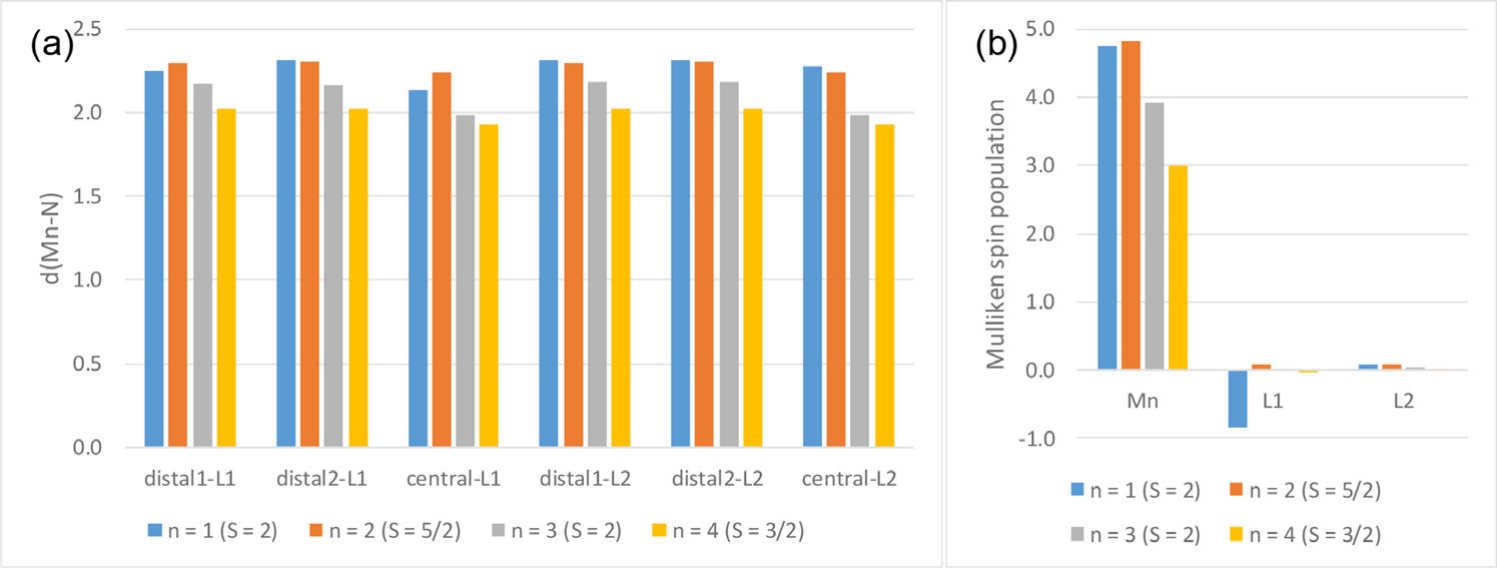
Graphical presentation of the average B3LYP/6-311G(d,p)/def2tzvpp calculated (a) Mn-N bond lengths and (b) Mulliken spin population on the Mn and the ligands, in [Mn(tpy)_2_]^n+^ complexes (1)–(6) in different oxidation states (*n* = 1, 2, 3 and 4). See Scheme 1 for definition of bonds, L1 and L2.

**Fig. 2:**
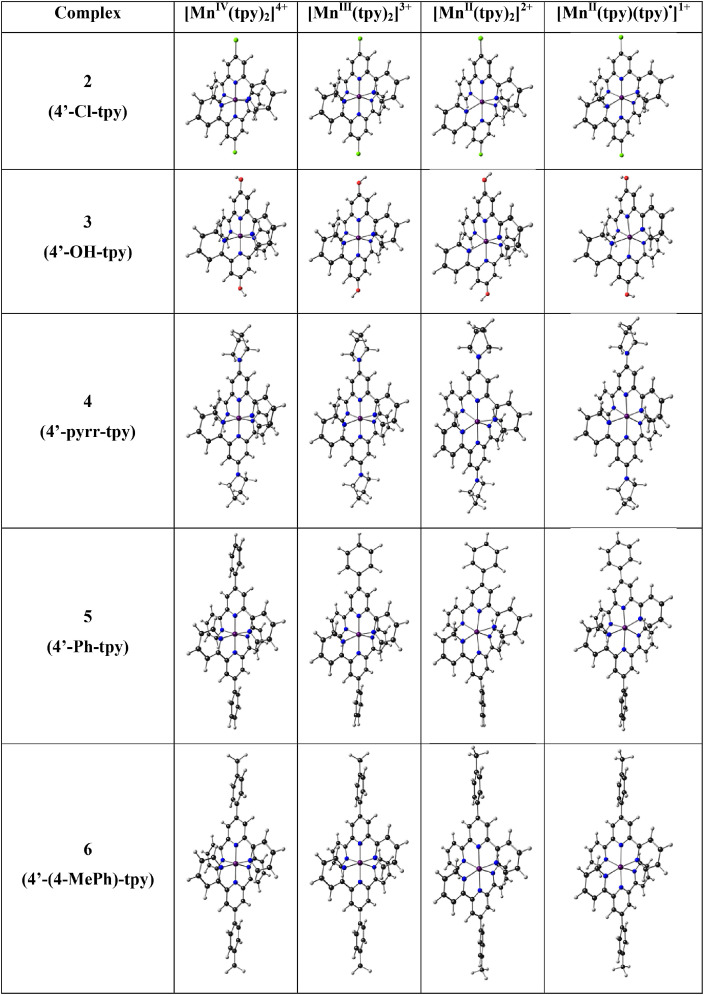
The B3LYP/6-311G(d,p)/def2tzvpp optimized geometry of the indicated [Mn(tpy)_3_]^n+^ in different oxidation states (*n* = 1, 2, 3 and 4). Color scheme used for atoms (online version): Mn (purple), N (blue), C (black), Cl (green) and H (white) (For interpretation of the references to color in this figure legend, the reader is referred to the web version of this article.).

**Fig. 3:**
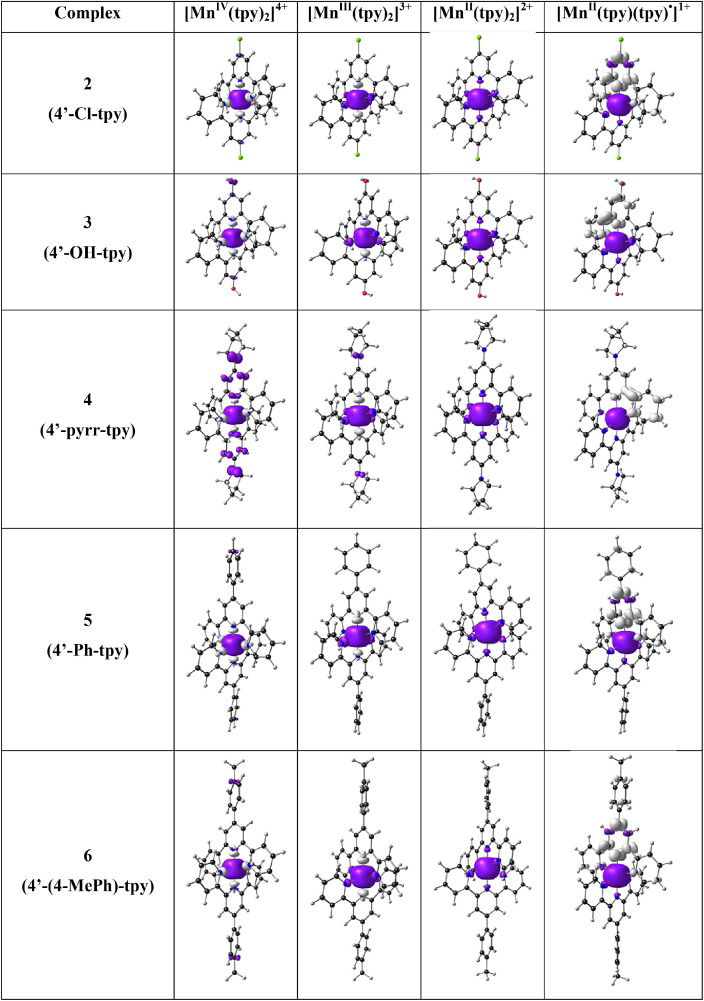
The B3LYP/6-311G(d,p)/def2tzvpp spin density plots of the indicated [Mn(tpy)_3_]^n+^ in different oxidation states (*n* = 1, 2, 3 and 4). A contour 0.004 Åe^−3^ was used for the spin plots. Color scheme used for atoms (online version): Mn (purple), N (blue), C (black), Cl (green) and H (white) (For interpretation of the references to color in this figure legend, the reader is referred to the web version of this article.).

**Fig. 4:**
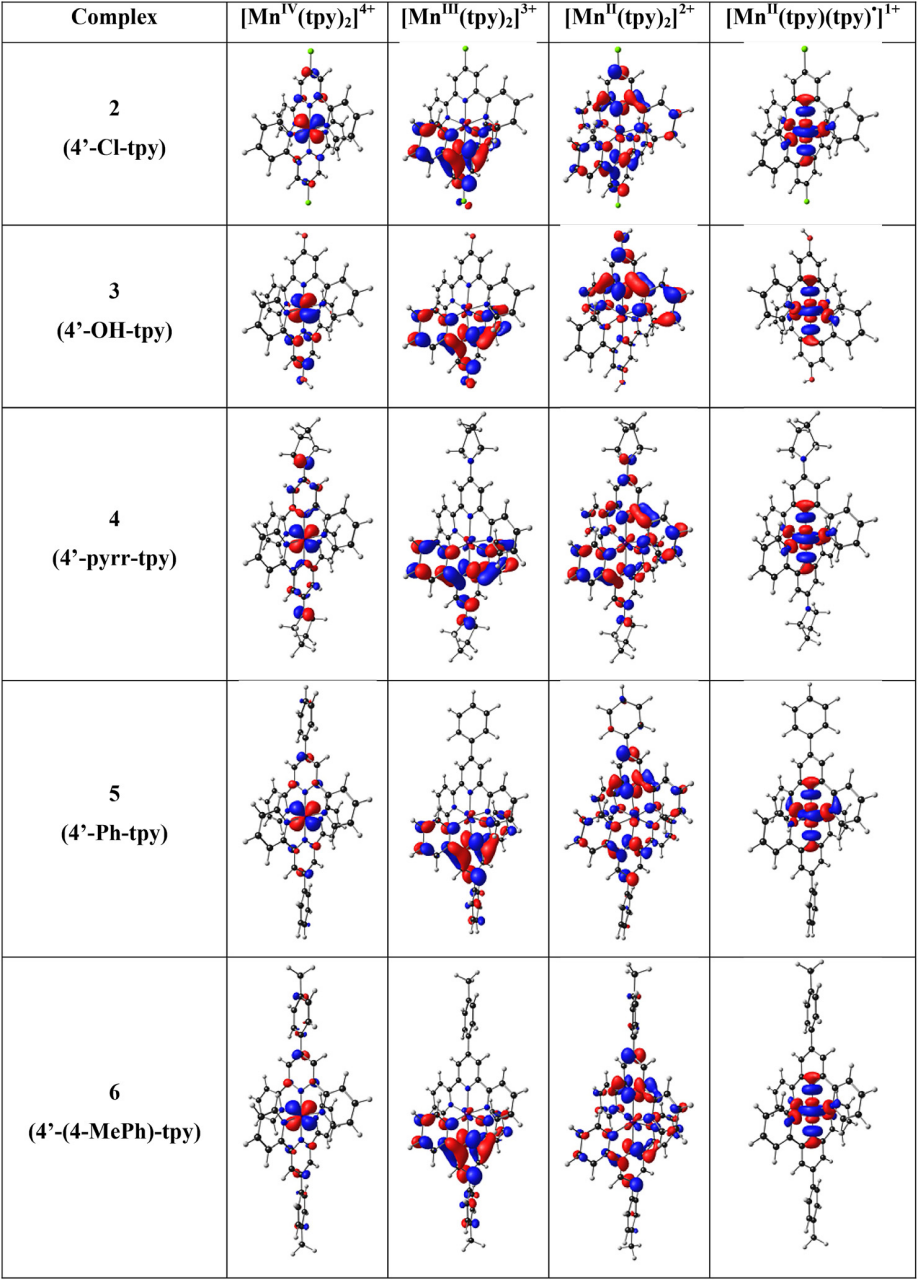
The B3LYP/6-311G(d,p)/def2tzvpp lowest unoccupied molecular orbitals of the indicated [Mn(tpy)_3_]^n+^ in different oxidation states (*n* = 1, 2, 3 and 4). A contour of 0.06 was used for the MO plots. Color scheme used for atoms (online version): Mn (purple), N (blue), C (black), Cl (green) and H (white) (For interpretation of the references to color in this figure legend, the reader is referred to the web version of this article.).

**Fig. 5:**
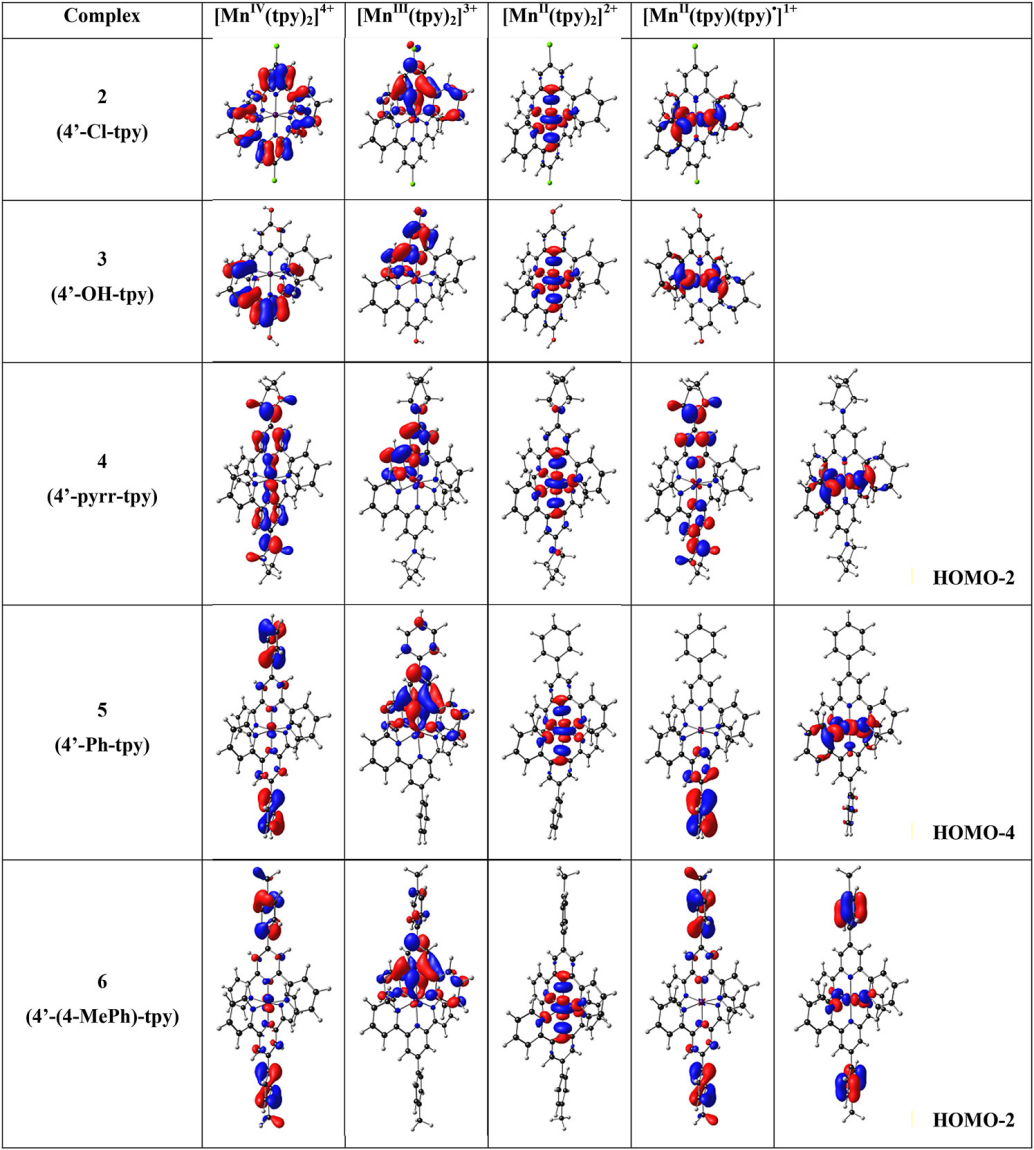
The B3LYP/6-311G(d,p)/def2tzvpp highest occupied molecular orbitals of the indicated [Mn(tpy)_3_]^n+^ in different oxidation states (*n* = 1, 2, 3 and 4). A contour of 0.06 was used for the MO plots. Color scheme used for atoms (online version): Mn (purple), N (blue), C (black), Cl (green) and H (white). The d_x2_-d_y2_ Mn-based MO is stabilized for [Mn^II^(tpy)(tpy)^•^]^1+^ of (4)–(6) (For interpretation of the references to color in this figure legend, the reader is referred to the web version of this article.).

In Fig. 1 the average Mn-N bond lengths and average Mulliken spin population (on Mn and on the two 4’-substituted 2,2’;6’2’’-terpyridine ligands) for complexes (1)–(6) in the different oxidation states (*n* = 1, 2, 3 and 4) are shown. [Mn^III^(tpy)_2_]^3+^ has a compression Jahn-Teller distortion geometry. In Fig. 2 the optimized geometries are presented for (2)–(6), with bond length data reported in Table 1 for (1)–(6). The spin plots of the *mono*-, *bis*-, *tris*- and *tetra*-cationic states of (2)–(6) are shown in Fig. 3 with selected Mulliken spin population data summarized in Table 2. Selected frontier orbitals of the *mono*-, *bis*-, *tris*- and *tetra*-cationic states of (2)–(6) are shown in Figs. 4 and 5. The Cartesian coordinates of all the structures of *mono*-, *bis*-, *tris*- and *tetra*-cationic states of [Mn(tpy)_2_]^n+^ complexes (1)–(6), are provided in the supplementary data files. The calculated structural (bond lengths and angles, coordinates of optimized geometries) and electronic (Mulliken spin population and character of frontier molecular orbitals) data reported, for the oxidation states 1, 2, 3 and 4, are obtained from the output files of files from the reduced bis(terpyridine)manganese(II) complexes (charge = 1) [10], the bis(terpyridine)manganese(II) complexes (charge = 2) [11], the bis(terpyridine)manganese(III) complexes (charge = 3) [12] and bis(terpyridine)manganese(IV) complexes (charge = 4) [13], respectively. For more insight into the provided data, see the related research article [5].

**Table 1:** Mn-N bond length data of *mono*-, *bis*-, *tris*- and *tetra*-cationic states of [Mn(tpy)_2_]^n+^ complexes (1)–(6).

		L1			L2		

	Mn-N bonds^a^ *n* = 1 (*S* = 2)	distal1-L1	distal2-L1	central-L1	distal1-L2	distal2-L2	central-L2
1	[Mn(tpy)_2_]^n+^	2.279	2.285	2.123	2.313	2.314	2.287
2	[Mn(4′-Cl-tpy)_2_]^n+^	2.274	2.297	2.124	2.313	2.313	2.289
3	[Mn(4′-OH-tpy)_2_]^n+^	2.199	2.362	2.164	2.321	2.318	2.276
4	[Mn(4′-pyrr-tpy)_2_]^n+^	2.189	2.371	2.176	2.328	2.330	2.259
5	[Mn(4′-Ph-tpy)_2_]^n+^	2.281	2.305	2.123	2.313	2.315	2.280
6	[Mn(4′-(4-MePh)-tpy)_2_]^n+^	2.299	2.281	2.123	2.314	2.315	2.276
	average	2.254	2.317	2.139	2.317	2.317	2.278
	MAD^b^	0.040	0.033	0.021	0.005	0.004	0.007

	***n* = 2 (*S* = 5/2)**						

1	[Mn(tpy)_2_]^n+^	2.299	2.299	2.241	2.299	2.299	2.241
2	[Mn(4′-Cl-tpy)_2_]^n+^	2.300	2.301	2.243	2.301	2.300	2.243
3	[Mn(4′-OH-tpy)_2_]^n+^	2.294	2.306	2.228	2.303	2.301	2.227
4	[Mn(4′-pyrr-tpy)_2_]^n+^	2.302	2.321	2.226	2.302	2.321	2.226
5	[Mn(4′-Ph-tpy)_2_]^n+^	2.297	2.318	2.251	2.294	2.320	2.252
6	[Mn(4′-(4-MePh)-tpy)_2_]^n+^	2.301	2.302	2.230	2.301	2.301	2.230
	average	2.299	2.308	2.236	2.300	2.307	2.236
	MAD^b^	0.002	0.008	0.009	0.002	0.009	0.009

	***n* = 3 (*S* = 2)**						

1	[Mn(tpy)_2_]^n+^	2.175	2.175	1.993	2.175	2.175	1.993
2	[Mn(4′-Cl-tpy)_2_]^n+^	2.181	2.181	1.995	2.181	2.181	1.995
3	[Mn(4′-OH-tpy)_2_]^n+^	2.190	2.190	1.989	2.162	2.163	1.978
4	[Mn(4′-pyrr-tpy)_2_]^n+^	2.178	2.177	1.967	2.178	2.177	1.967
5	[Mn(4′-Ph-tpy)_2_]^n+^	2.117	2.117	1.966	2.228	2.228	2.015
6	[Mn(4′-(4-MePh)-tpy)_2_]^n+^	2.175	2.175	1.984	2.176	2.175	1.985
	average	2.169	2.169	1.982	2.183	2.183	1.989
	MAD^b^	0.017	0.017	0.010	0.015	0.015	0.012

	***n* = 4 (S = 3/2)**						

1	[Mn(tpy)_2_]^n+^	2.022	2.022	1.941	2.022	2.022	1.941
2	[Mn(4′-Cl-tpy)_2_]^n+^	2.022	2.021	1.938	2.022	2.022	1.938
3	[Mn(4′-OH-tpy)_2_]^n+^	2.024	2.024	1.936	2.022	2.025	1.921
4	[Mn(4′-pyrr-tpy)_2_]^n+^	2.024	2.024	1.904	2.024	2.024	1.904
5	[Mn(4′-Ph-tpy)_2_]^n+^	2.022	2.022	1.925	2.022	2.021	1.925
6	[Mn(4′-(4-MePh)-tpy)_2_]^n+^	2.022	2.022	1.921	2.022	2.022	1.921
	average	2.023	2.022	1.927	2.022	2.023	1.925
	MAD^b^	0.001	0.001	0.011	0.000	0.001	0.010

a See Scheme 1 for definition of L1, L2 and different bonds.

b MAD = mean absolute deviation.

**Table 2:** Mulliken spin density population on Mn and the two ligands (L1 and L2) of *mono*-, *bis*-, *tris*- and *tetra*-cationic states of [Mn(tpy)_2_]^n+^ complexes (1)–(6).

	*n* = 1 (*S* = 2)	Mn	L1^a^	L2^a^
1	[Mn(tpy)_2_]^n+^	4.747	-0.825	0.078
2	[Mn(4′-Cl-tpy)_2_]^n+^	4.749	-0.827	0.078
3	[Mn(4′-OH-tpy)_2_]^n+^	4.766	-0.841	0.075
4	[Mn(4′-pyrr-tpy)_2_]^n+^	4.770	-0.843	0.073
5	[Mn(4′-Ph-tpy)_2_]^n+^	4.753	-0.831	0.078
6	[Mn(4′-(4-MePh)-tpy)_2_]^n+^	4.752	-0.830	0.078
	average	4.756	-0.833	0.077
	MAD^b^	0.008	0.006	0.002

	***n* = 2 (*S* = 5/2)**			

1	[Mn(tpy)_2_]^n+^	4.834	0.083	0.083
2	[Mn(4′-Cl-tpy)_2_]^n+^	4.835	0.083	0.083
3	[Mn(4′-OH-tpy)_2_]^n+^	4.832	0.084	0.084
4	[Mn(4′-pyrr-tpy)_2_]^n+^	4.831	0.084	0.084
5	[Mn(4′-Ph-tpy)_2_]^n+^	4.836	0.082	0.082
6	[Mn(4′-(4-MePh)-tpy)_2_]^n+^	4.832	0.084	0.084
	average	4.833	0.083	0.083
	MAD^b^	0.002	0.001	0.001

	***n* = 3 (*S* = 2)**			

1	[Mn(tpy)_2_]^n+^	3.951	0.024	0.024
2	[Mn(4′-Cl-tpy)_2_]^n+^	3.947	0.027	0.027
3	[Mn(4′-OH-tpy)_2_]^n+^	3.930	0.053	0.017
4	[Mn(4′-pyrr-tpy)_2_]^n+^	3.891	0.055	0.055
5	[Mn(4′-Ph-tpy)_2_]^n+^	3.935	-0.036	0.102
6	[Mn(4′-(4-MePh)-tpy)_2_]^n+^	3.931	0.034	0.035
	average	3.931	0.026	0.043
	MAD^b^	0.014	0.021	0.023

	***n* = 4 (*S* = 3/2)**			

1	[Mn(tpy)_2_]^n+^	3.143	-0.071	-0.071
2	[Mn(4′-Cl-tpy)_2_]^n+^	3.109	-0.054	-0.055
3	[Mn(4′-OH-tpy)_2_]^n+^	3.081	-0.054	-0.028
4	[Mn(4′-pyrr-tpy)_2_]^n+^	2.753	0.123	0.123
5	[Mn(4′-Ph-tpy)_2_]^n+^	3.000	0.000	0.000
6	[Mn(4′-(4-MePh)-tpy)_2_]^n+^	2.930	0.035	0.035
	average	3.003	-0.003	0.001
	MAD^b^	0.108	0.056	0.052

a See Scheme 1 for definition of L1 and L2.

b MAD = mean absolute deviation.

## 2 Experimental Design, Materials and Methods

Geometry optimizations and electronic structure calculations were done by density functional theory (DFT) calculations using the Gaussian 16 software program (Revision B.01) [14], similar to the computations described in the related research article [5]. The hybrid functional B3LYP [15,16] were used, while applying the GTO (Gaussian type orbital) triple-ζ basis set 6-311G(d,p) for the lighter atoms (C, H, N, F, O) and the def2-TZVPP basis set for both the core and valence electrons of Mn. Optimizations were performed in acetonitrile as implicit solvent using the Polarizable Continuum Model (PCM), which uses the integral equation formalism variant (IEFPCM). The Berny optimization algorithm [17] was used, requesting a convergence on energy of 1.0D-8 atomic unit. The input coordinates for the compounds were constructed using Chemcraft software [18]. The coordinates, charge and multiplicity were specified in the input files of the DFT calculations. If difficulty with convergence were experienced, the options opt=(tight), Int=(Grid=Ultrafine) and scf=(qc,maxcycle = 1000,tight,conver = 8) were specified in the input file. The geometrical parameters were obtained by visualizing the output files with the optimized structures in Chemcraft. Spin plots were obtained from cube files, generated with the cubegen keyword in Gaussian, and visualized in Chemcraft. Molecular orbital plots were generated in Chemcraft from the output files, with “gfinput POP(regular)” being specified in the input files. Many different input geometries with different Mn-N lengths, were optimized to ensure that the global minimum structure is indeed obtained, since many higher energy local minimum structures could also be optimized.

## Ethics Statements

This work does not require any ethical statement.

## CRediT authorship contribution statement

**Jeanet Conradie:** Conceptualization, Data curation, Methodology, Writing – review & editing.

## Declaration of Competing Interest

The authors declare that they have no known competing financial interests or personal relationships that could have appeared to influence the work reported in this paper.

## Data Availability

Reduced bis(terpyridine)manganese(II) complexes (Original data) (figshare), Bis(terpyridine)manganese(II) complexes (Original data) (figshare), Bis(terpyridine)manganese(III) complexes (Original data) (figshare), Bis(terpyridine)manganese(IV) complexes (Original data) (figshare).

## Appendix Supplementary materials

Image, application 1
