# Supplementary material for: Electronic and structural data of 4’-substituted bis(2,2’;6’2’’-terpyridine)manganese in mono-, bis-, tris- and tetra-cationic states from DFT calculations
Source: Data Brief. 2022 Apr 30;42:108221. doi: 10.1016/j.dib.2022.108221 (PMC9092895; doi:10.1016/j.dib.2022.108221)
Supplement: Supplementary file 1 [file mmc1.docx]

**Electronic and structural data of 4’-substituted bis(2,2’;6’2’’-terpyridine)manganese in *mono*-, *bis*-,** ***tris*- and** ***tetra*-cationic states from DFT calculations.**

**Authors**

Jeanet Conradie*

**Affiliations**

Department of Chemistry, University of the Free State, P.O. Box 339, Bloemfontein, 9300, South Africa; Department of Chemistry, UiT - The Arctic University of Norway, N-9037 Tromsø, Norway

**Corresponding author’s email address and Twitter handle**

Jeanet Conradie ([conradj@ufs.ac.za](mailto:conradj@ufs.ac.za))

@ConradieJeanet

Supporting information

**Optimized Cartesian coordinates (Å)**

All compounds were optimized with the B3LYP functional and the 6-311G(d,p) (C, H, N, F, O) and def2-TZVPP (Mn) basis set.

**Table of Contents**

[1. [Mn(tpy)_2_]^1+^, *S* = 2 2](#_Toc89029861)

[2. [Mn(4'-Cl-tpy)_2_]^1+^, *S* = 2 3](#_Toc89029862)

[3. [Mn(4'-OH-tpy)_2_]^1+^, *S* = 2 4](#_Toc89029863)

[4. [Mn(4'-pyrr-tpy)_2_]^1+^, *S* = 2 5](#_Toc89029864)

[5. [Mn(4'-Ph-tpy)_2_]^1+^, *S* = 2 7](#_Toc89029865)

[6. [Mn(4'-(4-MePh)-tpy)_2_]^1+^, *S* = 2 9](#_Toc89029866)

[7. [Mn(tpy)_2_]^2+^, *S = 5/2* 11](#_Toc89029867)

[8. [Mn(4'-Cl-tpy)_2_]^2+^, *S = 5/2* 12](#_Toc89029868)

[9. [Mn(4'-OH-tpy)_2_]^2+^, *S = 5/2* 13](#_Toc89029869)

[10. [Mn(4'-pyrr-tpy)_2_]^2+^, *S = 5/2* 14](#_Toc89029870)

[11. [Mn(4'-Ph-tpy)_2_]^2+^, *S = 5/2* 16](#_Toc89029871)

[12. [Mn(4'-(4-MePh)-tpy)_2_]^2+^, *S = 5/2* 18](#_Toc89029872)

[13. [Mn(tpy)_2_]^3+^, *S* = 2 20](#_Toc89029873)

[14. [Mn(4'-Cl-tpy)_2_]^3+^, *S* = 2 21](#_Toc89029874)

[15. [Mn(4'-OH-tpy)_2_]^3+^, *S* = 2 22](#_Toc89029875)

[16. [Mn(4'-pyrr-tpy)_2_]^3+^, *S* = 2 24](#_Toc89029876)

[17. [Mn(4'-Ph-tpy)_2_]^3+^, *S* = 2 25](#_Toc89029877)

[18. [Mn(4'-(4-MePh)-tpy)_2_]^3+^, *S* = 2 27](#_Toc89029878)

[19. [Mn(tpy)_2_]^4+^, *S = 3/2* 29](#_Toc89029879)

[20. [Mn(4'-Cl-tpy)_2_]^4+^, *S = 3/2* 30](#_Toc89029880)

[21. [Mn(4'-OH-tpy)_2_]^4+^, *S = 3/2* 31](#_Toc89029881)

[22. [Mn(4'-pyrr-tpy)_2_]^4+^, *S = 3/2* 33](#_Toc89029882)

[23. [Mn(4-Ph-tpy)_2_]^4+^, *S = 3/2* 34](#_Toc89029883)

[24. [Mn(4'-(4-MePh)-tpy)_2_]^4+^, *S = 3/2* 36](#_Toc89029884)

# [Mn(tpy)_2_]^1+^, *S* = 2

59

symmetry c1

Mn -0.057564000 0.019771000 0.013909000

N -0.713363000 1.567583000 1.553317000

C -2.063779000 1.723153000 1.680497000

C -2.587770000 2.648011000 2.605037000

C -1.733831000 3.402738000 3.385190000

C -0.346379000 3.235100000 3.244396000

C 0.104253000 2.310696000 2.321063000

N -2.180741000 0.031124000 -0.013148000

C -2.883855000 0.881546000 0.813049000

C -4.274262000 0.913176000 0.796447000

C -4.979381000 0.071736000 -0.067841000

C -4.264780000 -0.792148000 -0.903610000

C -2.875404000 -0.800802000 -0.863874000

N -0.697719000 -1.531319000 -1.537798000

C -2.045594000 -1.667423000 -1.699127000

C -2.560006000 -2.598438000 -2.622127000

C -1.697422000 -3.376190000 -3.370162000

C -0.312224000 -3.225867000 -3.196998000

C 0.128473000 -2.296081000 -2.273820000

N 0.703194000 -1.529501000 1.554172000

C 2.034283000 -1.687691000 1.696424000

C 2.561187000 -2.633617000 2.578159000

C 1.692818000 -3.421380000 3.326127000

C 0.321332000 -3.248764000 3.177369000

C -0.126621000 -2.289289000 2.276174000

N 2.229433000 0.007330000 0.010611000

C 2.887408000 -0.799032000 0.857845000

C 4.282377000 -0.781290000 0.930937000

C 4.981607000 0.089558000 0.104608000

C 4.289741000 0.917202000 -0.770850000

C 2.894558000 0.850205000 -0.794471000

N 0.716705000 1.554292000 -1.535659000

C 2.048968000 1.690776000 -1.688379000

C 2.583633000 2.568587000 -2.633610000

C 1.721721000 3.318529000 -3.426600000

C 0.348905000 3.175602000 -3.259703000

C -0.106854000 2.278104000 -2.300399000

H -3.657427000 2.769768000 2.709019000

H -2.133404000 4.114857000 4.097459000

H 0.357387000 3.806030000 3.835886000

H 1.167438000 2.146319000 2.178511000

H -4.816580000 1.586448000 1.446865000

H -4.800077000 -1.447256000 -1.577903000

H -3.628396000 -2.709907000 -2.747951000

H -2.089263000 -4.093827000 -4.081178000

H 0.397789000 -3.815045000 -3.762568000

H 1.189832000 -2.145694000 -2.104632000

H 3.628780000 -2.763955000 2.683364000

H 2.086122000 -4.159833000 4.013876000

H -0.388902000 -3.839993000 3.740158000

H -1.185950000 -2.118314000 2.121703000

H 4.817906000 -1.421774000 1.616382000

H 4.831007000 1.598997000 -1.410417000

H 3.652199000 2.669318000 -2.759433000

H 2.121030000 4.003267000 -4.164631000

H -0.356491000 3.740061000 -3.855127000

H -1.167497000 2.128131000 -2.133567000

H -6.061477000 0.088723000 -0.090682000

H 6.063095000 0.125257000 0.144901000

# [Mn(4'-Cl-tpy)_2_]^1+^, *S* = 2

59

symmetry c1

Mn -0.053780000 -0.049193000 -0.031209000

N -0.762533000 -1.905825000 -1.137019000

C -2.116700000 -2.056075000 -1.218765000

C -2.670882000 -3.174088000 -1.872177000

C -1.840672000 -4.122596000 -2.436184000

C -0.448806000 -3.956605000 -2.348654000

C 0.031590000 -2.839116000 -1.692667000

N -2.177189000 0.006943000 0.002605000

C -2.907238000 -1.000516000 -0.593595000

C -4.297959000 -0.988892000 -0.587489000

C -4.955161000 0.066597000 0.039242000

C -4.231640000 1.095066000 0.649673000

C -2.846117000 1.041187000 0.616273000

N -0.650414000 1.858588000 1.100088000

C -1.990343000 2.064544000 1.222576000

C -2.479409000 3.199472000 1.894151000

C -1.593060000 4.112366000 2.434997000

C -0.215140000 3.890259000 2.303775000

C 0.198737000 2.754780000 1.630821000

N 0.719784000 1.084007000 -1.893211000

C 2.049923000 1.248451000 -2.038453000

C 2.579799000 1.979866000 -3.102876000

C 1.713852000 2.540959000 -4.035659000

C 0.343769000 2.360369000 -3.884076000

C -0.107033000 1.624957000 -2.793259000

N 2.233586000 0.013652000 0.009687000

C 2.896640000 0.603361000 -0.996011000

C 4.291672000 0.595854000 -1.048363000

C 4.973733000 -0.043213000 -0.022028000

C 4.289524000 -0.655449000 1.018969000

C 2.894774000 -0.604205000 0.999014000

N 0.716318000 -1.169722000 1.839994000

C 2.046065000 -1.228501000 2.052010000

C 2.574497000 -1.836098000 3.192154000

C 1.707275000 -2.397057000 4.123635000

C 0.337253000 -2.337109000 3.896492000

C -0.111855000 -1.710373000 2.739164000

H -3.743839000 -3.295500000 -1.934321000

H -2.262538000 -4.984288000 -2.939566000

H 0.236678000 -4.675953000 -2.777079000

H 1.099235000 -2.669106000 -1.599244000

H -4.868931000 -1.776847000 -1.056631000

H -4.753626000 1.907399000 1.133434000

H -3.543884000 3.364275000 1.990282000

H -1.963731000 4.988724000 2.953027000

H 0.512484000 4.579572000 2.711600000

H 1.254805000 2.540696000 1.503992000

H 3.645973000 2.120537000 -3.210049000

H 2.108519000 3.111322000 -4.867347000

H -0.364370000 2.777727000 -4.587615000

H -1.165753000 1.460487000 -2.628270000

H 4.837891000 1.058739000 -1.855506000

H 4.833375000 -1.155536000 1.805258000

H 3.640785000 -1.870938000 3.363798000

H 2.100627000 -2.871273000 5.014238000

H -0.372156000 -2.760331000 4.595195000

H -1.170572000 -1.634558000 2.518937000

Cl -6.716242000 0.108768000 0.064189000

Cl 6.717662000 -0.083355000 -0.044487000

# [Mn(4'-OH-tpy)_2_]^1+^, *S* = 2

61

symmetry c1

Mn -0.037028000 0.174758000 0.145803000

N -0.782165000 1.656635000 1.589798000

C -2.162531000 1.754483000 1.706749000

C -2.726400000 2.681639000 2.622823000

C -1.917771000 3.481648000 3.392042000

C -0.508941000 3.372375000 3.261727000

C -0.013812000 2.456822000 2.359036000

N -2.195515000 0.055862000 0.038217000

C -2.927542000 0.884972000 0.864419000

C -4.345483000 0.835859000 0.836121000

C -4.978103000 -0.037022000 -0.018818000

C -4.213151000 -0.879101000 -0.863810000

C -2.837085000 -0.793937000 -0.796738000

N -0.620782000 -1.444359000 -1.471827000

C -1.945704000 -1.634545000 -1.643624000

C -2.424749000 -2.569298000 -2.567673000

C -1.522985000 -3.311438000 -3.318682000

C -0.156832000 -3.108416000 -3.134642000

C 0.243171000 -2.164011000 -2.199451000

N 0.700574000 -1.462484000 1.616737000

C 2.019724000 -1.738044000 1.648302000

C 2.528623000 -2.753900000 2.459548000

C 1.655686000 -3.488710000 3.254586000

C 0.297215000 -3.194758000 3.221021000

C -0.133778000 -2.172035000 2.383449000

N 2.219008000 -0.054735000 -0.047058000

C 2.877559000 -0.896836000 0.764911000

C 4.267881000 -0.964714000 0.777682000

C 4.984496000 -0.128357000 -0.081512000

C 4.291380000 0.746842000 -0.922076000

C 2.903258000 0.754855000 -0.875175000

N 0.746106000 1.637216000 -1.472975000

C 2.070729000 1.656232000 -1.722422000

C 2.608992000 2.472918000 -2.718770000

C 1.760073000 3.285750000 -3.461865000

C 0.395527000 3.264539000 -3.195415000

C -0.065124000 2.421460000 -2.190555000

H -3.802181000 2.760077000 2.716208000

H -2.352373000 4.188040000 4.089837000

H 0.164988000 3.983662000 3.847676000

H 1.058540000 2.341479000 2.228396000

H -4.932877000 1.478582000 1.480114000

H -4.727940000 -1.556232000 -1.529053000

H -3.486652000 -2.718103000 -2.701891000

H -1.882162000 -4.038004000 -4.037495000

H 0.580415000 -3.664843000 -3.698635000

H 1.294938000 -1.970696000 -2.020206000

H 3.585117000 -2.981377000 2.472251000

H 2.034920000 -4.280738000 3.888489000

H -0.416115000 -3.741313000 3.823678000

H -1.183065000 -1.907087000 2.319353000

H 4.796939000 -1.634812000 1.441244000

H 4.855024000 1.398236000 -1.573553000

H 3.670233000 2.476083000 -2.923124000

H 2.162281000 3.924086000 -4.238884000

H -0.299996000 3.881178000 -3.749287000

H -1.120128000 2.367558000 -1.946951000

O -6.331182000 -0.156140000 -0.117860000

H -6.758742000 0.456501000 0.492947000

O 6.326920000 -0.110234000 -0.142999000

H 6.708277000 -0.744209000 0.478233000

# [Mn(4'-pyrr-tpy)_2_]^1+^, *S* = 2

83

symmetry c1

Mn -0.035687000 0.347029000 -0.024261000

N 0.925425000 1.806384000 1.514169000

N 2.200952000 0.033745000 0.004807000

N 0.529334000 -1.331022000 -1.539066000

N 6.346996000 -0.286840000 -0.208752000

N -0.848999000 1.832770000 -1.410274000

N -2.195704000 0.099873000 0.055416000

N -0.567777000 -1.269083000 1.627256000

N -6.361632000 -0.314807000 0.087776000

C 0.206327000 2.642772000 2.271532000

H -0.866137000 2.628255000 2.113037000

C 0.777785000 3.490511000 3.212256000

H 0.154527000 4.149541000 3.802266000

C 2.159909000 3.460855000 3.367411000

H 2.649170000 4.101672000 4.090781000

C 2.912369000 2.595114000 2.582745000

H 3.985869000 2.562224000 2.702022000

C 2.265611000 1.773954000 1.655073000

C 2.989878000 0.815940000 0.767482000

C 4.372015000 0.741258000 0.729904000

H 4.970938000 1.382155000 1.356637000

C 5.004511000 -0.183264000 -0.139106000

C 4.152593000 -0.988425000 -0.936375000

H 4.578804000 -1.688583000 -1.636812000

C 2.778853000 -0.850244000 -0.832147000

C 1.830511000 -1.662680000 -1.650505000

C 2.239493000 -2.708128000 -2.482713000

H 3.281600000 -2.984213000 -2.558433000

C 1.286778000 -3.407461000 -3.214471000

H 1.589896000 -4.220107000 -3.863379000

C -0.053388000 -3.052577000 -3.099084000

H -0.826592000 -3.570493000 -3.651340000

C -0.383493000 -2.007394000 -2.244776000

H -1.413024000 -1.694120000 -2.112927000

C 7.279258000 0.581292000 0.531154000

H 7.267917000 0.330833000 1.598695000

H 6.998333000 1.631563000 0.424484000

C 8.639569000 0.274053000 -0.104711000

H 8.796768000 0.918358000 -0.974064000

H 9.462591000 0.437847000 0.591471000

C 8.503816000 -1.189602000 -0.547391000

H 8.651256000 -1.857296000 0.305850000

H 9.214821000 -1.471146000 -1.324610000

C 7.053621000 -1.280971000 -1.034951000

H 6.969229000 -1.023466000 -2.097497000

H 6.628379000 -2.276907000 -0.890934000

C -0.122199000 2.708246000 -2.138739000

H 0.952325000 2.666547000 -1.980949000

C -0.655373000 3.610701000 -3.031483000

H -0.010323000 4.284056000 -3.581144000

C -2.067986000 3.623750000 -3.201510000

H -2.533279000 4.316057000 -3.893771000

C -2.835487000 2.746980000 -2.475590000

H -3.911511000 2.751059000 -2.597601000

C -2.235359000 1.836241000 -1.565942000

C -2.965313000 0.897115000 -0.763528000

C -4.373901000 0.761101000 -0.771179000

H -4.964337000 1.376515000 -1.432923000

C -5.005315000 -0.162260000 0.064686000

C -4.172829000 -0.954861000 0.919551000

H -4.619745000 -1.657900000 1.603743000

C -2.803853000 -0.786851000 0.870586000

C -1.877032000 -1.576816000 1.733983000

C -2.308236000 -2.581494000 2.607483000

H -3.355961000 -2.834992000 2.683592000

C -1.376922000 -3.262765000 3.380341000

H -1.700469000 -4.041596000 4.060475000

C -0.028178000 -2.933726000 3.266479000

H 0.730743000 -3.439395000 3.849353000

C 0.325193000 -1.931655000 2.373072000

H 1.362497000 -1.643873000 2.241710000

C -7.277660000 0.519342000 -0.695121000

H -7.195545000 0.291479000 -1.767011000

H -7.047137000 1.581156000 -0.564481000

C -8.662377000 0.153070000 -0.147634000

H -9.449175000 0.286624000 -0.891314000

H -8.900437000 0.784204000 0.713462000

C -8.491705000 -1.306348000 0.297863000

H -9.235879000 -1.623161000 1.029898000

H -8.561051000 -1.973520000 -0.566128000

C -7.067903000 -1.339451000 0.867095000

H -7.061778000 -1.094643000 1.937771000

H -6.599967000 -2.320082000 0.745669000

# [Mn(4'-Ph-tpy)_2_]^1+^, *S* = 2

79

symmetry c1

Mn 0.048752000 0.173807000 -0.004575000

N -0.783180000 1.092705000 1.948329000

N -2.225194000 0.007258000 -0.000729000

N -0.656536000 -0.781140000 -1.991941000

N 0.827619000 2.126535000 -0.888910000

N 2.168085000 0.051985000 0.030188000

N 0.592901000 -1.848128000 0.958729000

C 0.010009000 1.596584000 2.899468000

H 1.072737000 1.595289000 2.685246000

C -0.478084000 2.097488000 4.101182000

H 0.203228000 2.496692000 4.840853000

C -1.851098000 2.065150000 4.317380000

H -2.274972000 2.440541000 5.240628000

C -2.681316000 1.541665000 3.332118000

H -3.749226000 1.507543000 3.493640000

C -2.116286000 1.062640000 2.148605000

C -2.927015000 0.491962000 1.034909000

C -4.320272000 0.469117000 1.039163000

H -4.875175000 0.890719000 1.864304000

C -5.009504000 -0.070082000 -0.053498000

C -4.250688000 -0.563656000 -1.121004000

H -4.750104000 -0.996627000 -1.975109000

C -2.859341000 -0.506916000 -1.065240000

C -1.976489000 -0.997463000 -2.161992000

C -2.463905000 -1.639784000 -3.301800000

H -3.521478000 -1.820792000 -3.430219000

C -1.568742000 -2.055942000 -4.281402000

H -1.931804000 -2.556006000 -5.170853000

C -0.209888000 -1.823561000 -4.101755000

H 0.520077000 -2.129933000 -4.839313000

C 0.199197000 -1.183451000 -2.937127000

H 1.247685000 -0.983979000 -2.747091000

C -6.491054000 -0.108700000 -0.082041000

C -7.221572000 -0.361291000 1.089007000

H -6.701163000 -0.554281000 2.019693000

C -8.612349000 -0.400656000 1.061503000

H -9.161259000 -0.608596000 1.972493000

C -9.296374000 -0.180551000 -0.133367000

H -10.379596000 -0.207962000 -0.153252000

C -8.581040000 0.074892000 -1.302597000

H -9.106044000 0.255257000 -2.233339000

C -7.189952000 0.106692000 -1.279377000

H -6.646878000 0.326843000 -2.190962000

C 0.064832000 3.142448000 -1.330908000

H -1.007669000 3.003928000 -1.241952000

C 0.583578000 4.305195000 -1.869892000

H -0.075800000 5.092562000 -2.210863000

C 1.978777000 4.424879000 -1.955630000

H 2.430671000 5.318394000 -2.369639000

C 2.775643000 3.389949000 -1.504097000

H 3.851772000 3.476844000 -1.564457000

C 2.183775000 2.231325000 -0.967228000

C 2.938338000 1.083807000 -0.457933000

C 4.322715000 1.019300000 -0.462794000

H 4.893926000 1.837516000 -0.878609000

C 4.999794000 -0.113855000 0.032001000

C 4.191620000 -1.159506000 0.530779000

H 4.662228000 -2.037850000 0.949614000

C 2.811307000 -1.058724000 0.524436000

C 1.925490000 -2.108540000 1.042080000

C 2.380538000 -3.319860000 1.593631000

H 3.439312000 -3.529239000 1.658705000

C 1.468701000 -4.250880000 2.055910000

H 1.814033000 -5.185220000 2.482242000

C 0.098302000 -3.971674000 1.965245000

H -0.648274000 -4.673073000 2.314180000

C -0.281881000 -2.762807000 1.410350000

H -1.330959000 -2.503112000 1.315593000

C 6.470790000 -0.204117000 0.028883000

C 7.275775000 0.949833000 0.106422000

H 6.811053000 1.924512000 0.199126000

C 8.664060000 0.862721000 0.101128000

H 9.255948000 1.768903000 0.169797000

C 9.295262000 -0.379259000 0.022111000

H 10.377012000 -0.446297000 0.019485000

C 8.515202000 -1.533712000 -0.053282000

H 8.990200000 -2.506122000 -0.124218000

C 7.126682000 -1.448614000 -0.051669000

H 6.544641000 -2.358427000 -0.141087000

# [Mn(4'-(4-MePh)-tpy)_2_]^1+^, *S* = 2

85

symmetry c1

Mn 0.049107000 -0.111294000 -0.065707000

N -0.735344000 -1.484053000 1.623334000

N -2.225369000 -0.031891000 -0.025033000

C 0.081934000 -2.175801000 2.424001000

H 1.142563000 -2.078797000 2.221530000

C -0.379712000 -2.977608000 3.461754000

H 0.320671000 -3.519185000 4.083759000

C -1.752046000 -3.056864000 3.670048000

H -2.156165000 -3.667748000 4.467874000

C -2.607310000 -2.339864000 2.840344000

H -3.675112000 -2.394854000 2.996799000

C -2.067428000 -1.557325000 1.817575000

C -2.904056000 -0.757834000 0.876998000

C -4.296445000 -0.746099000 0.916586000

H -4.829821000 -1.316388000 1.662488000

C -5.013335000 0.031400000 -0.001474000

C -6.493031000 0.066013000 0.013258000

C -7.238103000 -1.076298000 0.341195000

H -6.731818000 -2.008913000 0.561593000

C -8.627755000 -1.041551000 0.348724000

H -9.179292000 -1.944040000 0.589664000

C -9.326046000 0.132207000 0.041918000

C -10.832617000 0.175740000 0.085720000

H -11.182195000 0.478435000 1.078791000

H -11.265083000 -0.803681000 -0.128240000

H -11.231065000 0.894652000 -0.633160000

N 0.622108000 1.612550000 1.343538000

N -0.700917000 1.280008000 -1.756714000

N 0.795688000 -1.796319000 -1.410250000

N 2.169966000 -0.035877000 -0.012072000

C -0.239253000 2.408822000 1.999613000

C 0.133068000 1.915928000 -2.585864000

C 0.017198000 -2.654766000 -2.093018000

H -1.292176000 2.196581000 1.846282000

H 1.190083000 1.727044000 -2.436324000

H -1.053198000 -2.519058000 -1.976644000

C 0.158397000 3.443839000 2.826601000

C -0.307939000 2.773619000 -3.587322000

C 0.517008000 -3.658758000 -2.901398000

H -0.578055000 4.054613000 3.332300000

H 0.405222000 3.265827000 -4.235293000

H -0.155257000 -4.322475000 -3.429236000

C 1.533261000 3.669947000 2.981801000

C -1.676798000 2.974364000 -3.724705000

C 1.910412000 -3.783387000 -3.009741000

H 1.892003000 4.470314000 3.617985000

H -2.065016000 3.634817000 -4.490284000

H 2.347921000 -4.555562000 -3.631319000

C 2.431531000 2.859621000 2.312407000

C -2.549276000 2.316279000 -2.864651000

C 2.723740000 -2.909439000 -2.313706000

H 3.493430000 3.029604000 2.425360000

H -3.614574000 2.468175000 -2.963708000

H 3.798411000 -3.000642000 -2.391807000

C 1.959375000 1.821399000 1.488852000

C -2.029725000 1.468745000 -1.884049000

C 2.150640000 -1.907363000 -1.508318000

C 2.829686000 0.908525000 0.739204000

C -2.885618000 0.719889000 -0.919490000

C 2.923517000 -0.935877000 -0.731457000

C -4.277642000 0.772929000 -0.934315000

C 4.309060000 -0.903502000 -0.712150000

C 4.212132000 0.971994000 0.779087000

H -4.796958000 1.362249000 -1.675315000

H 4.868452000 -1.610015000 -1.309433000

H 4.696168000 1.711382000 1.401565000

C 5.003782000 0.059665000 0.047527000

C 6.476517000 0.111820000 0.076569000

C -7.190543000 1.241345000 -0.303034000

C 7.258304000 -1.042407000 -0.121246000

C 7.166131000 1.316949000 0.307250000

H -6.645926000 2.147497000 -0.541548000

H 6.775211000 -2.001433000 -0.269071000

H 6.611182000 2.238113000 0.443033000

C -8.580022000 1.271046000 -0.284374000

C 8.647062000 -0.989630000 -0.093486000

C 8.555690000 1.361959000 0.336495000

H -9.093777000 2.195901000 -0.524237000

H 9.213538000 -1.904334000 -0.238642000

H 9.049874000 2.312344000 0.512811000

C 9.328410000 0.212593000 0.133529000

C 10.836340000 0.270129000 0.132943000

H 11.203803000 1.095591000 0.746698000

H 11.270360000 -0.658020000 0.512426000

H 11.225039000 0.419074000 -0.880849000

# [Mn(tpy)_2_]^2+^, *S = 5/2*

59

symmetry c1

Mn 0.000000000 -0.000027000 0.000009000

N -0.715987000 1.544757000 1.544143000

C -2.050052000 1.691284000 1.690076000

C -2.581485000 2.604407000 2.601849000

C -1.718912000 3.375933000 3.372913000

C -0.346807000 3.219402000 3.217213000

C 0.108471000 2.290269000 2.289204000

N -2.240598000 -0.000322000 -0.000731000

C -2.900970000 0.826454000 0.825976000

C -4.296822000 0.849397000 0.849596000

C -4.991741000 -0.001471000 -0.000824000

C -4.296056000 -0.851713000 -0.851246000

C -2.900222000 -0.827569000 -0.827567000

N -0.714600000 -1.544822000 -1.544862000

C -2.048533000 -1.691636000 -1.691670000

C -2.579169000 -2.604290000 -2.604375000

C -1.715930000 -3.375196000 -3.375311000

C -0.343963000 -3.218496000 -3.218583000

C 0.110507000 -2.289744000 -2.289798000

N 0.714879000 -1.544357000 1.545187000

C 2.048839000 -1.690730000 1.692204000

C 2.579634000 -2.602601000 2.605599000

C 1.716527000 -3.373325000 3.376866000

C 0.344533000 -3.217187000 3.219815000

C -0.110099000 -2.289092000 2.290452000

N 2.240598000 0.000008000 0.000656000

C 2.900372000 -0.826986000 0.827625000

C 4.296210000 -0.851210000 0.850971000

C 4.991741000 -0.001088000 0.000304000

C 4.296668000 0.849657000 -0.850114000

C 2.900819000 0.826597000 -0.826358000

N 0.715708000 1.544687000 -1.544354000

C 2.049746000 1.691114000 -1.690619000

C 2.581021000 2.603834000 -2.602887000

C 1.718315000 3.375143000 -3.374020000

C 0.346238000 3.218773000 -3.217919000

C -0.108880000 2.289979000 -2.289493000

H -3.649650000 2.719445000 2.716021000

H -2.117600000 4.088388000 4.084323000

H 0.360026000 3.798635000 3.796134000

H 1.169982000 2.136268000 2.135704000

H -4.837604000 1.510215000 1.510756000

H -6.074433000 -0.001974000 -0.000807000

H -4.836257000 -1.513113000 -1.512304000

H -3.647233000 -2.719330000 -2.719461000

H -2.113997000 -4.087282000 -4.087438000

H 0.363372000 -3.797258000 -3.797365000

H 1.171890000 -2.135548000 -2.135595000

H 3.647718000 -2.717090000 2.721054000

H 2.114718000 -4.084806000 4.089528000

H -0.362703000 -3.795834000 3.798833000

H -1.171506000 -2.135253000 2.136071000

H 4.836527000 -1.512628000 1.511915000

H 6.074433000 -0.001594000 0.000089000

H 4.837334000 1.510462000 -1.511384000

H 3.649166000 2.718678000 -2.717435000

H 2.116880000 4.087286000 -4.085811000

H -0.360696000 3.797850000 -3.796875000

H -1.170365000 2.136064000 -2.135729000

# [Mn(4'-Cl-tpy)_2_]^2+^, *S = 5/2*

59

symmetry c1

Mn -0.000013000 0.004262000 -0.000194000

N -0.755466000 1.574287000 1.501967000

C -2.092050000 1.721797000 1.620797000

C -2.644003000 2.667660000 2.484317000

C -1.797593000 3.470472000 3.241952000

C -0.422980000 3.310342000 3.119164000

C 0.052524000 2.348935000 2.234529000

N -2.242414000 0.007728000 -0.050776000

C -2.921248000 0.820097000 0.773833000

C -4.315169000 0.801925000 0.819787000

C -4.979582000 -0.083163000 -0.018737000

C -4.277758000 -0.919826000 -0.876337000

C -2.885004000 -0.843386000 -0.865445000

N -0.688318000 -1.535352000 -1.564898000

C -2.018814000 -1.685262000 -1.736851000

C -2.532781000 -2.574643000 -2.680252000

C -1.654438000 -3.325717000 -3.454266000

C -0.286290000 -3.170444000 -3.269164000

C 0.150848000 -2.261231000 -2.312546000

N 0.688870000 -1.533270000 1.566715000

C 2.019387000 -1.683474000 1.738044000

C 2.533624000 -2.572395000 2.681733000

C 1.655451000 -3.322604000 3.456787000

C 0.287243000 -3.166930000 3.272433000

C -0.150126000 -2.258282000 2.315380000

N 2.242360000 0.008349000 0.050763000

C 2.885266000 -0.842280000 0.865687000

C 4.277998000 -0.918830000 0.876031000

C 4.979528000 -0.082803000 0.017567000

C 4.314821000 0.801895000 -0.821136000

C 2.920914000 0.820274000 -0.774533000

N 0.754932000 1.574437000 -1.502254000

C 2.091505000 1.721729000 -1.621546000

C 2.643288000 2.667253000 -2.485545000

C 1.796742000 3.469942000 -3.243149000

C 0.422147000 3.310026000 -3.119885000

C -0.053203000 2.348939000 -2.234823000

H -3.714222000 2.788372000 2.570346000

H -2.211151000 4.209716000 3.916428000

H 0.271693000 3.912339000 3.689475000

H 1.117044000 2.192896000 2.106847000

H -4.874853000 1.442753000 1.483150000

H -4.808761000 -1.605256000 -1.518333000

H -3.598218000 -2.684953000 -2.821504000

H -2.038411000 -4.020002000 -4.191120000

H 0.432147000 -3.734315000 -3.848908000

H 1.209261000 -2.107242000 -2.139423000

H 3.599109000 -2.683082000 2.822378000

H 2.039608000 -4.016527000 4.193888000

H -0.431045000 -3.730040000 3.853101000

H -1.208574000 -2.103892000 2.142801000

H 4.809208000 -1.603846000 1.518296000

H 4.874277000 1.442286000 -1.485115000

H 3.713491000 2.787796000 -2.571997000

H 2.210188000 4.208928000 -3.917977000

H -0.272618000 3.911912000 -3.690198000

H -1.117698000 2.193014000 -2.106877000

Cl -6.720575000 -0.147114000 0.007748000

Cl 6.720493000 -0.147047000 -0.009746000

# [Mn(4'-OH-tpy)_2_]^2+^, *S = 5/2*

61

symmetry c1

Mn 0.000623000 -0.039870000 0.025730000

N 0.767213000 -1.495983000 1.623778000

N 2.227242000 0.016849000 -0.001091000

N 0.665709000 1.466007000 -1.589626000

N -0.710751000 1.580917000 1.499854000

N -2.226256000 -0.002154000 -0.007145000

N -0.720389000 -1.636446000 -1.465729000

O 6.329815000 -0.043703000 -0.088079000

H 6.703171000 0.553397000 -0.749800000

O -6.326943000 -0.037082000 0.152504000

H -6.722484000 -0.699782000 -0.428820000

C -0.034437000 -2.211153000 2.421788000

H -1.100173000 -2.086430000 2.268876000

C 0.449820000 -3.072168000 3.398979000

H -0.238089000 -3.630118000 4.020078000

C 1.826825000 -3.188642000 3.549179000

H 2.247734000 -3.846032000 4.299712000

C 2.665176000 -2.448155000 2.723031000

H 3.736750000 -2.529663000 2.835037000

C 2.105102000 -1.606080000 1.761905000

C 2.924519000 -0.776413000 0.833568000

C 4.311478000 -0.808118000 0.816262000

H 4.887392000 -1.441714000 1.474110000

C 4.989273000 0.009084000 -0.093533000

C 4.257538000 0.830150000 -0.955744000

H 4.775652000 1.459450000 -1.665771000

C 2.869321000 0.806909000 -0.877574000

C 1.994109000 1.641855000 -1.749042000

C 2.496266000 2.555765000 -2.675945000

H 3.560134000 2.702067000 -2.794578000

C 1.609204000 3.292480000 -3.453199000

H 1.984821000 4.005437000 -4.176538000

C 0.242517000 3.101739000 -3.287552000

H -0.482105000 3.653057000 -3.871756000

C -0.182570000 2.177366000 -2.340695000

H -1.238521000 1.998152000 -2.174628000

C 0.114877000 2.366331000 2.201422000

H 1.175490000 2.180025000 2.079738000

C -0.338697000 3.372164000 3.046249000

H 0.368411000 3.981262000 3.593356000

C -1.710207000 3.567230000 3.160211000

H -2.107267000 4.342375000 3.803734000

C -2.573702000 2.754137000 2.434504000

H -3.641338000 2.901012000 2.512270000

C -2.043612000 1.761313000 1.609823000

C -2.893695000 0.843600000 0.799619000

C -4.279671000 0.844077000 0.864263000

H -4.831627000 1.510037000 1.510568000

C -4.988342000 -0.060186000 0.067488000

C -4.287371000 -0.936277000 -0.765832000

H -4.828764000 -1.644884000 -1.376992000

C -2.898186000 -0.875133000 -0.776287000

C -2.054381000 -1.762593000 -1.626568000

C -2.589355000 -2.670726000 -2.540679000

H -3.657479000 -2.762001000 -2.675582000

C -1.730028000 -3.463890000 -3.293160000

H -2.131358000 -4.172753000 -4.006633000

C -0.357575000 -3.332332000 -3.117968000

H 0.346325000 -3.929518000 -3.682044000

C 0.101228000 -2.402566000 -2.192549000

H 1.162868000 -2.263309000 -2.025060000

# [Mn(4'-pyrr-tpy)_2_]^2+^, *S = 5/2*

83

symmetry c1

Mn 0.000018000 0.298727000 0.000132000

N 0.909197000 1.826362000 1.462756000

N 2.211203000 0.046619000 -0.009278000

N 0.554475000 -1.315233000 -1.573687000

N 6.354492000 -0.289539000 -0.147944000

N -0.908788000 1.826004000 -1.463035000

N -2.211214000 0.047175000 0.009693000

N -0.554834000 -1.314318000 1.574658000

N -6.354516000 -0.289322000 0.146998000

C 0.175542000 2.676593000 2.192078000

H -0.894781000 2.653760000 2.021743000

C 0.731745000 3.547707000 3.119814000

H 0.098052000 4.217828000 3.685415000

C 2.111302000 3.525806000 3.295212000

H 2.588126000 4.183987000 4.011086000

C 2.878082000 2.645810000 2.540669000

H 3.949843000 2.619821000 2.675089000

C 2.248737000 1.803036000 1.621922000

C 2.988611000 0.834647000 0.760464000

C 4.369380000 0.756449000 0.745995000

H 4.959507000 1.400040000 1.378120000

C 5.013594000 -0.177417000 -0.106038000

C 4.172519000 -0.980826000 -0.918567000

H 4.609403000 -1.683039000 -1.610051000

C 2.798910000 -0.838424000 -0.839090000

C 1.859571000 -1.640719000 -1.676726000

C 2.280844000 -2.669604000 -2.522166000

H 3.324774000 -2.939577000 -2.591383000

C 1.340011000 -3.359332000 -3.277647000

H 1.654174000 -4.158938000 -3.937198000

C -0.002286000 -3.011453000 -3.171993000

H -0.767390000 -3.521563000 -3.742170000

C -0.346726000 -1.984085000 -2.302673000

H -1.380540000 -1.683090000 -2.179553000

C 7.278174000 0.574686000 0.608896000

H 7.240743000 0.327527000 1.676345000

H 7.007664000 1.626383000 0.491766000

C 8.649260000 0.253410000 0.004263000

H 8.830661000 0.891927000 -0.864604000

H 9.457845000 0.414356000 0.717691000

C 8.511507000 -1.211467000 -0.433470000

H 8.634053000 -1.875705000 0.426291000

H 9.237302000 -1.502933000 -1.193055000

C 7.072270000 -1.293803000 -0.953578000

H 7.013854000 -1.039405000 -2.018364000

H 6.634989000 -2.285005000 -0.814344000

C -0.174939000 2.675930000 -2.192516000

H 0.895375000 2.652915000 -2.022146000

C -0.730934000 3.546966000 -3.120453000

H -0.097084000 4.216810000 -3.686205000

C -2.110492000 3.525340000 -3.295851000

H -2.587169000 4.183487000 -4.011855000

C -2.877479000 2.645676000 -2.541129000

H -3.949250000 2.619959000 -2.675519000

C -2.248331000 1.802925000 -1.622229000

C -2.988420000 0.834820000 -0.760640000

C -4.369188000 0.756498000 -0.746639000

H -4.959152000 1.399757000 -1.379252000

C -5.013613000 -0.177118000 0.105505000

C -4.172749000 -0.980192000 0.918589000

H -4.609828000 -1.682314000 1.610041000

C -2.799132000 -0.837627000 0.839614000

C -1.859999000 -1.639461000 1.677921000

C -2.281496000 -2.667590000 2.524162000

H -3.325490000 -2.937228000 2.593691000

C -1.340804000 -3.356971000 3.280141000

H -1.655153000 -4.155988000 3.940318000

C 0.001570000 -3.009492000 3.174194000

H 0.766570000 -3.519355000 3.744732000

C 0.346225000 -1.982825000 2.304126000

H 1.380094000 -1.682102000 2.180807000

C -7.277994000 0.574579000 -0.610462000

H -7.240190000 0.327050000 -1.677811000

H -7.007576000 1.626331000 -0.493606000

C -8.649263000 0.253430000 -0.006176000

H -9.457620000 0.414092000 -0.719927000

H -8.830987000 0.892229000 0.862416000

C -8.511575000 -1.211296000 0.432084000

H -9.237602000 -1.502547000 1.191530000

H -8.633813000 -1.875821000 -0.427498000

C -7.072499000 -1.293397000 0.952684000

H -7.014441000 -1.038693000 2.017416000

H -6.635123000 -2.284616000 0.813871000

# [Mn(4'-Ph-tpy)_2_]^2+^, *S = 5/2*

79

symmetry c1

Mn 0.000630000 0.314102000 -0.003580000

N -0.911764000 1.852971000 1.432843000

N -2.237103000 0.060656000 -0.011531000

N -0.562568000 -1.324055000 -1.546846000

N 0.913394000 1.814240000 -1.483801000

N 2.237030000 0.060673000 0.009160000

N 0.561816000 -1.276835000 1.585848000

C -0.175853000 2.717115000 2.142407000

H 0.894435000 2.687772000 1.974179000

C -0.730971000 3.613002000 3.048185000

H -0.094759000 4.294257000 3.597241000

C -2.109939000 3.602156000 3.222667000

H -2.584644000 4.280172000 3.920992000

C -2.880238000 2.707060000 2.488357000

H -3.952394000 2.689276000 2.620736000

C -2.251977000 1.841279000 1.592265000

C -2.996607000 0.858988000 0.755642000

C -4.386005000 0.768187000 0.739182000

H -4.987486000 1.435556000 1.338143000

C -5.011630000 -0.172216000 -0.088197000

C -4.193687000 -0.982750000 -0.884184000

H -4.643295000 -1.724263000 -1.527457000

C -2.809367000 -0.838411000 -0.827383000

C -1.868835000 -1.647511000 -1.652427000

C -2.294039000 -2.677650000 -2.492578000

H -3.339517000 -2.939630000 -2.566721000

C -1.353845000 -3.378354000 -3.239816000

H -1.669839000 -4.180616000 -3.894978000

C -0.011415000 -3.035578000 -3.131746000

H 0.753608000 -3.552769000 -3.695409000

C 0.336125000 -2.001936000 -2.269934000

H 1.371598000 -1.706064000 -2.151169000

C -6.487172000 -0.299467000 -0.124357000

C -7.246294000 -0.133657000 1.044150000

H -6.751498000 0.065305000 1.987499000

C -8.631829000 -0.257305000 1.011413000

H -9.202655000 -0.137924000 1.924784000

C -9.282237000 -0.539752000 -0.189112000

H -10.361714000 -0.632278000 -0.214285000

C -8.538499000 -0.702808000 -1.357175000

H -9.037955000 -0.914102000 -2.295390000

C -7.152121000 -0.588344000 -1.325792000

H -6.588316000 -0.696493000 -2.244853000

C 0.177321000 2.653140000 -2.222858000

H -0.892899000 2.630748000 -2.052960000

C 0.732141000 3.514300000 -3.161952000

H 0.095887000 4.174932000 -3.735606000

C 2.110907000 3.495955000 -3.337396000

H 2.585351000 4.147352000 -4.060789000

C 2.881360000 2.627413000 -2.572038000

H 3.953410000 2.604662000 -2.704474000

C 2.253249000 1.793997000 -1.645663000

C 2.997425000 0.838931000 -0.777450000

C 4.386566000 0.742855000 -0.762778000

H 4.986491000 1.358055000 -1.416626000

C 5.011573000 -0.172360000 0.092798000

C 4.192776000 -0.956490000 0.914282000

H 4.643253000 -1.644415000 1.614121000

C 2.808467000 -0.817210000 0.848540000

C 1.867877000 -1.598142000 1.700134000

C 2.292920000 -2.603041000 2.570404000

H 3.337980000 -2.865334000 2.649431000

C 1.353108000 -3.278025000 3.341413000

H 1.669125000 -4.059345000 4.021394000

C 0.010949000 -2.937114000 3.224488000

H -0.753829000 -3.434912000 3.805668000

C -0.336748000 -1.931496000 2.330226000

H -1.372194000 -1.639006000 2.202912000

C 6.486764000 -0.302346000 0.133160000

C 7.309732000 0.822883000 -0.026618000

H 6.867404000 1.803407000 -0.156596000

C 8.694925000 0.698347000 0.015194000

H 9.316123000 1.579040000 -0.098023000

C 9.281030000 -0.551944000 0.208503000

H 10.360073000 -0.648267000 0.237654000

C 8.473392000 -1.677828000 0.363893000

H 8.921875000 -2.654090000 0.505722000

C 7.087800000 -1.554884000 0.330968000

H 6.472416000 -2.441076000 0.431763000

# [Mn(4'-(4-MePh)-tpy)_2_]^2+^, *S = 5/2*

85

symmetry c1

Mn 0.000001000 0.004284000 0.000086000

N -0.715501000 -1.546398000 1.542897000

N -2.229784000 0.003423000 0.001196000

C 0.108304000 -2.297238000 2.283342000

H 1.169708000 -2.127473000 2.145853000

C -0.347403000 -3.249908000 3.186732000

H 0.358595000 -3.832059000 3.763782000

C -1.719542000 -3.427771000 3.318741000

H -2.118725000 -4.161625000 4.007814000

C -2.581348000 -2.650760000 2.552447000

H -3.649214000 -2.785998000 2.645651000

C -2.049605000 -1.709476000 1.669841000

C -2.897461000 -0.832458000 0.813350000

C -4.289072000 -0.852596000 0.840785000

H -4.814995000 -1.503102000 1.523378000

C -5.016463000 -0.001509000 -0.001339000

C -6.495538000 -0.005063000 -0.002514000

C -7.213548000 -1.193421000 0.199249000

H -6.685584000 -2.130605000 0.331619000

C -8.603236000 -1.194477000 0.190341000

H -9.133562000 -2.130147000 0.331287000

C -9.328635000 -0.013395000 -0.006945000

C -10.835998000 -0.013166000 0.020074000

H -11.204052000 0.148464000 1.039095000

H -11.239981000 -0.967023000 -0.325082000

H -11.245086000 0.783090000 -0.604969000

N 0.718237000 1.555696000 1.540452000

N -0.718202000 1.555600000 -1.540460000

N 0.715466000 -1.546307000 -1.542894000

N 2.229787000 0.003458000 -0.001172000

C -0.104383000 2.308826000 2.279869000

C 0.104429000 2.308713000 -2.279879000

C -0.108352000 -2.297106000 -2.283365000

H -1.166040000 2.140547000 2.142506000

H 1.166084000 2.140374000 -2.142564000

H -1.169754000 -2.127335000 -2.145864000

C 0.352840000 3.261987000 3.181979000

C -0.352775000 3.261931000 -3.181940000

C 0.347335000 -3.249741000 -3.186802000

H -0.352235000 3.846055000 3.758220000

H 0.352313000 3.845985000 -3.758181000

H -0.358676000 -3.831858000 -3.763871000

C 1.725266000 3.437827000 3.313763000

C -1.725195000 3.437846000 -3.313673000

C 1.719471000 -3.427616000 -3.318831000

H 2.125613000 4.171994000 4.001827000

H -2.125527000 4.172061000 -4.001694000

H 2.118639000 -4.161445000 -4.007939000

C 2.585850000 2.658402000 2.548533000

C -2.585793000 2.658432000 -2.548447000

C 2.581291000 -2.650647000 -2.552512000

H 3.653933000 2.792065000 2.641519000

H -3.653872000 2.792149000 -2.641396000

H 3.649156000 -2.785893000 -2.645731000

C 2.052608000 1.716791000 1.667202000

C -2.052569000 1.716764000 -1.667166000

C 2.049566000 -1.709395000 -1.669860000

C 2.899000000 0.837204000 0.811896000

C -2.898979000 0.837185000 -0.811870000

C 2.897442000 -0.832414000 -0.813352000

C -4.290646000 0.852516000 -0.841797000

C 4.289053000 -0.852566000 -0.840803000

C 4.290668000 0.852520000 0.841807000

H -4.817555000 1.501047000 -1.525500000

H 4.814962000 -1.503064000 -1.523416000

H 4.817596000 1.501038000 1.525508000

C 5.016463000 -0.001505000 0.001329000

C 6.495537000 -0.005079000 0.002486000

C -7.219825000 1.178525000 -0.209380000

C 7.213526000 -1.193451000 -0.199276000

C 7.219844000 1.178500000 0.209328000

H -6.697024000 2.117780000 -0.347435000

H 6.685542000 -2.130626000 -0.331627000

H 6.697059000 2.117765000 0.347383000

C -8.609600000 1.171081000 -0.206830000

C 8.603213000 -1.194528000 -0.190390000

C 8.609620000 1.171034000 0.206758000

H -9.145110000 2.101957000 -0.359659000

H 9.133527000 -2.130203000 -0.331349000

H 9.145143000 2.101907000 0.359556000

C 9.328633000 -0.013457000 0.006880000

C 10.835999000 -0.013254000 -0.020092000

H 11.245075000 0.782779000 0.605244000

H 11.239944000 -0.967231000 0.324772000

H 11.204106000 0.148718000 -1.039039000

# [Mn(tpy)_2_]^3+^, *S* = 2

59

symmetry c1

Mn 0.000000000 0.000000000 0.000000000

N 0.000000000 -2.123680000 0.468073000

N 0.000000000 2.123680000 0.468073000

N 0.000000000 0.000000000 1.993017000

N 0.000000000 0.000000000 -1.993017000

N -2.123680000 0.000000000 -0.468073000

N 2.123680000 0.000000000 -0.468073000

C 0.000000000 -3.143227000 -0.399841000

H 0.000000000 -2.892566000 -1.452426000

C 0.000000000 -2.382636000 1.798783000

C 0.000000000 2.382636000 1.798783000

C 0.000000000 3.143227000 -0.399841000

H 0.000000000 2.892566000 -1.452426000

C 0.000000000 4.469460000 0.015631000

C 0.000000000 4.741881000 1.377708000

C 0.000000000 3.685471000 2.283998000

C 0.000000000 -4.469460000 0.015631000

C 0.000000000 -3.685471000 2.283998000

C 0.000000000 -4.741881000 1.377708000

H 0.000000000 5.261133000 -0.720976000

H 0.000000000 5.762977000 1.736721000

H 0.000000000 -5.762977000 1.736721000

H 0.000000000 -5.261133000 -0.720976000

H 0.000000000 -3.881592000 3.346476000

H 0.000000000 3.881592000 3.346476000

C 0.000000000 -1.184715000 2.656516000

C 0.000000000 1.184715000 2.656516000

C 0.000000000 1.204575000 4.046664000

C 0.000000000 0.000000000 4.739821000

C 0.000000000 -1.204575000 4.046664000

H 0.000000000 -2.142169000 4.582321000

H 0.000000000 0.000000000 5.822011000

H 0.000000000 2.142169000 4.582321000

C -3.143227000 0.000000000 0.399841000

H -2.892566000 0.000000000 1.452426000

C -2.382636000 0.000000000 -1.798783000

C 2.382636000 0.000000000 -1.798783000

C 3.143227000 0.000000000 0.399841000

H 2.892566000 0.000000000 1.452426000

C 4.469460000 0.000000000 -0.015631000

C 4.741881000 0.000000000 -1.377708000

C 3.685471000 0.000000000 -2.283998000

C -4.469460000 0.000000000 -0.015631000

C -3.685471000 0.000000000 -2.283998000

C -4.741881000 0.000000000 -1.377708000

H 5.261133000 0.000000000 0.720976000

H 5.762977000 0.000000000 -1.736721000

H -5.762977000 0.000000000 -1.736721000

H -5.261133000 0.000000000 0.720976000

H -3.881592000 0.000000000 -3.346476000

H 3.881592000 0.000000000 -3.346476000

C -1.184715000 0.000000000 -2.656516000

C 1.184715000 0.000000000 -2.656516000

C 1.204575000 0.000000000 -4.046664000

C 0.000000000 0.000000000 -4.739821000

C -1.204575000 0.000000000 -4.046664000

H -2.142169000 0.000000000 -4.582321000

H 0.000000000 0.000000000 -5.822011000

H 2.142169000 0.000000000 -4.582321000

# [Mn(4'-Cl-tpy)_2_]^3+^, *S* = 2

59

symmetry c1

Mn -0.000005000 -0.000002000 0.000025000

N -0.479233000 0.866104000 1.943533000

C -1.810089000 0.963935000 2.181576000

C -2.299183000 1.482658000 3.374655000

C -1.395412000 1.909060000 4.343873000

C -0.033382000 1.807601000 4.093842000

C 0.385481000 1.279953000 2.877788000

N -1.994663000 0.000002000 -0.000047000

C -2.662424000 0.477631000 1.082479000

C -4.049614000 0.488172000 1.106613000

C -4.737306000 -0.000105000 -0.000093000

C -4.049541000 -0.488319000 -1.106780000

C -2.662351000 -0.477657000 -1.082604000

N -0.479107000 -0.866085000 -1.943543000

C -1.809946000 -0.963872000 -2.181685000

C -2.298972000 -1.482441000 -3.374859000

C -1.395147000 -1.908764000 -4.344059000

C -0.033131000 -1.807382000 -4.093916000

C 0.385660000 -1.279868000 -2.877778000

N 0.479152000 -1.943561000 0.866125000

C 1.810000000 -2.181632000 0.963978000

C 2.299063000 -3.374722000 1.482709000

C 1.395267000 -4.343923000 1.909095000

C 0.033243000 -4.093863000 1.807614000

C -0.385586000 -2.877799000 1.279961000

N 1.994657000 -0.000004000 0.000058000

C 2.662374000 -1.082556000 0.477689000

C 4.049564000 -1.106740000 0.488243000

C 4.737302000 -0.000060000 -0.000027000

C 4.049582000 1.106654000 -0.488245000

C 2.662392000 1.082529000 -0.477596000

N 0.479185000 1.943534000 -0.866068000

C 1.810033000 2.181639000 -0.963823000

C 2.299103000 3.374802000 -1.482373000

C 1.395314000 4.344030000 -1.908711000

C 0.033289000 4.093923000 -1.807363000

C -0.385548000 2.877794000 -1.279864000

H -3.361764000 1.556715000 3.555206000

H -1.756891000 2.314575000 5.279961000

H 0.701617000 2.127965000 4.819333000

H 1.438501000 1.188862000 2.648004000

H -4.589717000 0.865457000 1.961087000

H -4.589590000 -0.865673000 -1.961259000

H -3.361542000 -1.556416000 -3.555505000

H -1.756572000 -2.314157000 -5.280221000

H 0.701910000 -2.127692000 -4.819388000

H 1.438668000 -1.188837000 -2.647909000

H 3.361638000 -3.555294000 1.556786000

H 1.756720000 -5.280016000 2.314619000

H -0.701776000 -4.819339000 2.127967000

H -1.438601000 -2.647993000 1.188857000

H 4.589633000 -1.961235000 0.865527000

H 4.589665000 1.961112000 -0.865598000

H 3.361680000 3.555422000 -1.556316000

H 1.756774000 5.280185000 -2.314086000

H -0.701725000 4.819418000 -2.127685000

H -1.438563000 2.647953000 -1.188852000

Cl -6.464350000 -0.000187000 -0.000114000

Cl 6.464346000 -0.000105000 -0.000098000

# [Mn(4'-OH-tpy)_2_]^3+^, *S* = 2

61

symmetry c1

Mn -0.012889000 -0.002054000 0.001651000

N 0.475270000 -2.103288000 0.380536000

N 1.976101000 0.001074000 -0.003523000

N 0.468089000 2.099562000 -0.383347000

N -0.470351000 0.378126000 2.080589000

N -1.990961000 -0.000824000 0.005279000

N -0.480742000 -0.382654000 -2.075233000

O 6.067499000 -0.068884000 0.005000000

H 6.480152000 0.793563000 -0.145587000

O -6.078258000 0.024132000 0.089226000

H -6.494830000 -0.129505000 -0.770811000

C -0.388232000 -3.109938000 0.565176000

H -1.441700000 -2.865076000 0.530114000

C 0.033128000 -4.414300000 0.791946000

H -0.698968000 -5.197384000 0.935111000

C 1.397004000 -4.676966000 0.827585000

H 1.761336000 -5.681622000 1.000651000

C 2.297844000 -3.633448000 0.637802000

H 3.361070000 -3.824120000 0.663430000

C 1.805469000 -2.351859000 0.415320000

C 2.655470000 -1.163720000 0.199586000

C 4.034223000 -1.188692000 0.201958000

H 4.587676000 -2.101658000 0.363521000

C 4.740929000 0.004231000 -0.007636000

C 4.032085000 1.195193000 -0.215714000

H 4.565254000 2.120501000 -0.379924000

C 2.650446000 1.165020000 -0.209186000

C 1.797492000 2.351675000 -0.423745000

C 2.285099000 3.634178000 -0.650720000

H 3.347263000 3.829658000 -0.681591000

C 1.380407000 4.674749000 -0.839515000

H 1.741430000 5.679852000 -1.016781000

C 0.017652000 4.408179000 -0.798429000

H -0.717375000 5.188584000 -0.941158000

C -0.398867000 3.102790000 -0.567335000

H -1.451448000 2.854450000 -0.528562000

C 0.409499000 0.558449000 3.074140000

H 1.458900000 0.516816000 2.814276000

C 0.007404000 0.789288000 4.383732000

H 0.751826000 0.928787000 5.155705000

C -1.352168000 0.834282000 4.666142000

H -1.700654000 1.011425000 5.675663000

C -2.269611000 0.648965000 3.636191000

H -3.330071000 0.681675000 3.840905000

C -1.798429000 0.421296000 2.348474000

C -2.663062000 0.208714000 1.172210000

C -4.041414000 0.217110000 1.202537000

H -4.591130000 0.382400000 2.117248000

C -4.752467000 0.006765000 0.011461000

C -4.049234000 -0.205817000 -1.182831000

H -4.586183000 -0.368494000 -2.106327000

C -2.667891000 -0.205329000 -1.156605000

C -1.810132000 -0.422823000 -2.337382000

C -2.287172000 -0.653421000 -3.622417000

H -3.348133000 -0.684404000 -3.824800000

C -1.374409000 -0.846013000 -4.655469000

H -1.727501000 -1.026158000 -5.662841000

C -0.013851000 -0.805055000 -4.378531000

H 0.727138000 -0.950233000 -5.152744000

C 0.394080000 -0.570545000 -3.071214000

H 1.444652000 -0.531416000 -2.815870000

# [Mn(4'-pyrr-tpy)_2_]^3+^, *S* = 2

83

symmetry c1

Mn -0.000024000 -0.007848000 -0.000032000

N 0.462178000 1.495526000 1.506420000

N 1.966770000 -0.008323000 0.014471000

N 0.482910000 -1.512548000 -1.497064000

N 6.110918000 0.032819000 0.001265000

N -0.462303000 1.494235000 -1.507679000

N -1.966792000 -0.008502000 -0.014532000

N -0.482785000 -1.511455000 1.498070000

N -6.110931000 0.032749000 -0.000950000

C -0.416769000 2.206376000 2.225256000

H -1.465572000 2.022350000 2.031349000

C -0.015487000 3.135474000 3.175376000

H -0.757577000 3.688874000 3.734467000

C 1.345899000 3.327254000 3.383534000

H 1.695885000 4.041269000 4.118169000

C 2.261260000 2.591376000 2.638889000

H 3.321238000 2.730171000 2.795885000

C 1.789680000 1.678491000 1.699781000

C 2.651853000 0.835983000 0.843599000

C 4.023210000 0.875412000 0.859877000

H 4.530778000 1.550514000 1.529220000

C 4.777566000 0.019929000 0.005239000

C 4.035022000 -0.850374000 -0.844735000

H 4.551200000 -1.507947000 -1.524826000

C 2.663355000 -0.840914000 -0.816964000

C 1.812808000 -1.703254000 -1.664831000

C 2.296637000 -2.645665000 -2.567819000

H 3.358507000 -2.801613000 -2.693008000

C 1.391083000 -3.395040000 -3.311032000

H 1.750478000 -4.132056000 -4.017882000

C 0.027168000 -3.188626000 -3.135755000

H -0.707449000 -3.751289000 -3.695440000

C -0.386649000 -2.236000000 -2.214763000

H -1.437695000 -2.043499000 -2.042822000

C 6.935761000 0.960807000 0.806596000

H 6.887625000 0.674347000 1.862317000

H 6.569066000 1.983388000 0.705435000

C 8.345171000 0.778781000 0.235604000

H 8.491610000 1.450219000 -0.614341000

H 9.113059000 0.996852000 0.977597000

C 8.354889000 -0.682804000 -0.232154000

H 8.508631000 -1.352452000 0.617952000

H 9.126557000 -0.890936000 -0.973079000

C 6.948696000 -0.882653000 -0.804994000

H 6.897894000 -0.595225000 -1.860282000

H 6.595221000 -1.910087000 -0.705858000

C 0.416593000 2.204563000 -2.227094000

H 1.465411000 2.020841000 -2.032981000

C 0.015238000 3.132755000 -3.178070000

H 0.757290000 3.685754000 -3.737608000

C -1.346160000 3.324141000 -3.386513000

H -1.696193000 4.037418000 -4.121841000

C -2.261468000 2.588813000 -2.641258000

H -3.321460000 2.727250000 -2.798482000

C -1.789819000 1.676882000 -1.701263000

C -2.651935000 0.835094000 -0.844328000

C -4.023291000 0.874538000 -0.860518000

H -4.530893000 1.549146000 -1.530334000

C -4.777580000 0.019810000 -0.005062000

C -4.034972000 -0.849799000 0.845571000

H -4.551099000 -1.506799000 1.526253000

C -2.663307000 -0.840409000 0.817645000

C -1.812668000 -1.702117000 1.666058000

C -2.296381000 -2.643957000 2.569702000

H -3.358234000 -2.799895000 2.695057000

C -1.390736000 -3.392794000 3.313347000

H -1.750044000 -4.129364000 4.020705000

C -0.026845000 -3.186429000 3.137835000

H 0.707841000 -3.748687000 3.697837000

C 0.386860000 -2.234395000 2.216181000

H 1.437886000 -2.041962000 2.044041000

C -6.935832000 0.960105000 -0.806952000

H -6.887825000 0.672774000 -1.862443000

H -6.569088000 1.982756000 -0.706675000

C -8.345184000 0.778603000 -0.235648000

H -9.113149000 0.996081000 -0.977738000

H -8.491506000 1.450760000 0.613749000

C -8.354906000 -0.682591000 0.233333000

H -9.126489000 -0.890067000 0.974531000

H -8.508789000 -1.352949000 -0.616188000

C -6.948651000 -0.882024000 0.806165000

H -6.897709000 -0.593733000 1.861209000

H -6.595230000 -1.909555000 0.707835000

# [Mn(4'-Ph-tpy)_2_]^3+^, *S* = 2

79

symmetry c1

Mn -0.049227000 0.000027000 -0.000751000

N 0.471617000 -2.053797000 -0.689668000

N 1.965328000 0.000206000 -0.000461000

N 0.470978000 2.054097000 0.687785000

N -0.478694000 0.677834000 -1.960112000

N -2.015695000 -0.000229000 -0.000200000

N -0.477275000 -0.678188000 1.958667000

C -0.375712000 -3.034505000 -1.024140000

H -1.433027000 -2.810663000 -0.961401000

C 0.063659000 -4.286807000 -1.436219000

H -0.656057000 -5.050471000 -1.697664000

C 1.431428000 -4.522524000 -1.501450000

H 1.811348000 -5.485311000 -1.818637000

C 2.315939000 -3.506036000 -1.154558000

H 3.381144000 -3.678575000 -1.203425000

C 1.803638000 -2.276293000 -0.750340000

C 2.642334000 -1.122393000 -0.360559000

C 4.027553000 -1.140990000 -0.364355000

H 4.548026000 -2.033367000 -0.676011000

C 4.756738000 0.000366000 0.000313000

C 4.027219000 1.141645000 0.364550000

H 4.547423000 2.034090000 0.676461000

C 2.641999000 1.122894000 0.359973000

C 1.802946000 2.276699000 0.749304000

C 2.314844000 3.506489000 1.153915000

H 3.380001000 3.679139000 1.203432000

C 1.429993000 4.522856000 1.500322000

H 1.809588000 5.485670000 1.817821000

C 0.062282000 4.287000000 1.434228000

H -0.657675000 5.050571000 1.695281000

C -0.376676000 3.034662000 1.021786000

H -1.433941000 2.810713000 0.958370000

C 6.231390000 0.000427000 0.000738000

C 6.946023000 -1.170778000 0.303126000

H 6.417823000 -2.079104000 0.566214000

C 8.335971000 -1.166619000 0.309519000

H 8.873898000 -2.073193000 0.559210000

C 9.034522000 0.000509000 0.001533000

H 10.118090000 0.000541000 0.001836000

C 8.336075000 1.167597000 -0.306843000

H 8.874091000 2.074203000 -0.556226000

C 6.946123000 1.171677000 -0.301239000

H 6.418025000 2.079978000 -0.564623000

C 0.416335000 1.007235000 -2.901268000

H 1.461328000 0.917840000 -2.637871000

C 0.031953000 1.445268000 -4.162131000

H 0.787865000 1.700255000 -4.891946000

C -1.323123000 1.543586000 -4.451470000

H -1.656187000 1.881583000 -5.424332000

C -2.256356000 1.202664000 -3.476383000

H -3.313817000 1.274235000 -3.686253000

C -1.806978000 0.771570000 -2.234983000

C -2.683458000 0.382168000 -1.117607000

C -4.067193000 0.390863000 -1.136911000

H -4.587258000 0.723150000 -2.022726000

C -4.793940000 -0.000209000 0.000812000

C -4.066369000 -0.391363000 1.137987000

H -4.585797000 -0.723654000 2.024178000

C -2.682652000 -0.382707000 1.117668000

C -1.805370000 -0.772258000 2.234356000

C -2.253864000 -1.203832000 3.475907000

H -3.311179000 -1.275676000 3.686413000

C -1.319949000 -1.544895000 4.450287000

H -1.652331000 -1.883303000 5.423240000

C 0.034924000 -1.446176000 4.160128000

H 0.791352000 -1.701216000 4.889388000

C 0.418419000 -1.007656000 2.899169000

H 1.463224000 -0.917856000 2.635199000

C -6.267624000 -0.000129000 0.001374000

C -6.982810000 -0.266940000 -1.178534000

H -6.455621000 -0.503305000 -2.094702000

C -8.372488000 -0.273219000 -1.173786000

H -8.910935000 -0.495747000 -2.087029000

C -9.070292000 0.000105000 0.002465000

H -10.153833000 0.000203000 0.002883000

C -8.371530000 0.273298000 1.178175000

H -8.909226000 0.495912000 2.091841000

C -6.981850000 0.266781000 1.181839000

H -6.453898000 0.503031000 2.097597000

# [Mn(4'-(4-MePh)-tpy)_2_]^3+^, *S* = 2

85

symmetry c1

Mn 0.000150000 0.002747000 0.000169000

N -0.474314000 -1.500660000 1.499352000

N -1.984353000 0.002632000 0.000659000

C 0.397031000 -2.220661000 2.216997000

H 1.448041000 -2.029084000 2.045060000

C -0.014100000 -3.172223000 3.141943000

H 0.723645000 -3.731223000 3.701014000

C -1.375895000 -3.381319000 3.321394000

H -1.732265000 -4.116844000 4.031088000

C -2.285253000 -2.635201000 2.577724000

H -3.346431000 -2.792415000 2.706008000

C -1.804330000 -1.695350000 1.672248000

C -2.659684000 -0.840875000 0.826284000

C -4.043043000 -0.852588000 0.842709000

H -4.561323000 -1.504075000 1.529261000

C -4.775069000 -0.000272000 -0.001394000

C -6.245121000 -0.003041000 -0.002524000

C -6.964650000 -1.166873000 0.317836000

H -6.441522000 -2.088776000 0.540787000

C -8.352295000 -1.167953000 0.305929000

H -8.883611000 -2.084437000 0.536786000

C -9.076283000 -0.010437000 -0.007337000

C -10.581813000 -0.005317000 0.017613000

H -10.946693000 0.328069000 0.995260000

H -10.986391000 -1.003094000 -0.160836000

H -10.989419000 0.676414000 -0.731496000

N 0.475711000 1.505152000 1.499371000

N -0.475271000 1.506348000 -1.498355000

N 0.473865000 -1.499880000 -1.499534000

N 1.984487000 0.002177000 -0.000391000

C -0.395195000 2.225584000 2.217106000

C 0.395683000 2.227055000 -2.215745000

C -0.397794000 -2.219343000 -2.217354000

H -1.446306000 2.034391000 2.045385000

H 1.446775000 2.035585000 -2.044182000

H -1.448734000 -2.027645000 -2.045134000

C 0.016571000 3.177072000 3.141855000

C -0.015993000 3.179133000 -3.139928000

C 0.012933000 -3.170537000 -3.142851000

H -0.720814000 3.736454000 3.701017000

H 0.721433000 3.738725000 -3.698830000

H -0.725068000 -3.729092000 -3.702030000

C 1.378493000 3.385650000 3.320956000

C -1.377900000 3.388018000 -3.318806000

C 1.374636000 -3.379857000 -3.322704000

H 1.735308000 4.121179000 4.030421000

H -1.734661000 4.124008000 -4.027821000

H 1.730689000 -4.115117000 -4.032830000

C 2.287394000 2.639013000 2.577228000

C -2.286854000 2.641084000 -2.575444000

C 2.284321000 -2.634303000 -2.578870000

H 3.348688000 2.795822000 2.705109000

H -3.348134000 2.798126000 -2.703152000

H 3.345435000 -2.791693000 -2.707453000

C 1.805881000 1.699224000 1.672037000

C -1.805400000 1.700696000 -1.670830000

C 1.803829000 -1.694786000 -1.672832000

C 2.660628000 0.844042000 0.826204000

C -2.660275000 0.845123000 -0.825493000

C 2.659543000 -0.840935000 -0.826637000

C -4.043702000 0.854186000 -0.843848000

C 4.042889000 -0.852744000 -0.843383000

C 4.044056000 0.852886000 0.844362000

H -4.562147000 1.504507000 -1.531375000

H 4.560975000 -1.503803000 -1.530488000

H 4.562693000 1.502721000 1.532201000

C 4.775173000 -0.001064000 0.001156000

C 6.245220000 -0.003885000 0.001920000

C -6.969840000 1.156798000 -0.327905000

C 6.964606000 -1.167092000 -0.319940000

C 6.970118000 1.155844000 0.328406000

H -6.450570000 2.079733000 -0.555558000

H 6.441576000 -2.088793000 -0.543943000

H 6.450797000 2.078504000 0.557052000

C -8.356998000 1.150225000 -0.322669000

C 8.352534000 -1.168072000 -0.308577000

C 8.356994000 1.149371000 0.322714000

H -8.892408000 2.061586000 -0.564742000

H 8.883754000 -2.084182000 -0.540981000

H 8.892432000 2.060609000 0.565344000

C 9.076409000 -0.011151000 0.005825000

C 10.581969000 -0.004373000 -0.017694000

H 10.988206000 0.626857000 0.775514000

H 10.987010000 -1.011243000 0.095479000

H 10.947843000 0.394637000 -0.969970000

# [Mn(tpy)_2_]^4+^, *S = 3/2*

59

symmetry c1

Mn 0.000000000 0.000000000 0.000000000

N 1.991375000 0.000000000 0.351381000

N -1.991375000 0.000000000 0.351381000

N 0.000000000 0.000000000 1.940575000

N 0.000000000 0.000000000 -1.940575000

N 0.000000000 -1.991375000 -0.351381000

N 0.000000000 1.991375000 -0.351381000

C 2.949354000 0.000000000 -0.591301000

H 2.640383000 0.000000000 -1.626135000

C 2.335191000 0.000000000 1.675765000

C -2.335191000 0.000000000 1.675765000

C -2.949354000 0.000000000 -0.591301000

H -2.640383000 0.000000000 -1.626135000

C -4.295267000 0.000000000 -0.253011000

C -4.657505000 0.000000000 1.087767000

C -3.664194000 0.000000000 2.064470000

C 4.295267000 0.000000000 -0.253011000

C 3.664194000 0.000000000 2.064470000

C 4.657505000 0.000000000 1.087767000

H -5.034480000 0.000000000 -1.041855000

H -5.700476000 0.000000000 1.376020000

H 5.700476000 0.000000000 1.376020000

H 5.034480000 0.000000000 -1.041855000

H 3.922218000 0.000000000 3.113709000

H -3.922218000 0.000000000 3.113709000

C 1.191572000 0.000000000 2.588605000

C -1.191572000 0.000000000 2.588605000

C -1.213359000 0.000000000 3.976383000

C 0.000000000 0.000000000 4.663189000

C 1.213359000 0.000000000 3.976383000

H 2.150105000 0.000000000 4.515116000

H 0.000000000 0.000000000 5.745255000

H -2.150105000 0.000000000 4.515116000

C 0.000000000 -2.949354000 0.591301000

H 0.000000000 -2.640383000 1.626135000

C 0.000000000 -2.335191000 -1.675765000

C 0.000000000 2.335191000 -1.675765000

C 0.000000000 2.949354000 0.591301000

H 0.000000000 2.640383000 1.626135000

C 0.000000000 4.295267000 0.253011000

C 0.000000000 4.657505000 -1.087767000

C 0.000000000 3.664194000 -2.064470000

C 0.000000000 -4.295267000 0.253011000

C 0.000000000 -3.664194000 -2.064470000

C 0.000000000 -4.657505000 -1.087767000

H 0.000000000 5.034480000 1.041855000

H 0.000000000 5.700476000 -1.376020000

H 0.000000000 -5.700476000 -1.376020000

H 0.000000000 -5.034480000 1.041855000

H 0.000000000 -3.922218000 -3.113709000

H 0.000000000 3.922218000 -3.113709000

C 0.000000000 -1.191572000 -2.588605000

C 0.000000000 1.191572000 -2.588605000

C 0.000000000 1.213359000 -3.976383000

C 0.000000000 0.000000000 -4.663189000

C 0.000000000 -1.213359000 -3.976383000

H 0.000000000 -2.150105000 -4.515116000

H 0.000000000 0.000000000 -5.745255000

H 0.000000000 2.150105000 -4.515116000

# [Mn(4'-Cl-tpy)_2_]^4+^, *S = 3/2*

59

symmetry c1

Mn -0.000118000 -0.002129000 0.000432000

N -0.356933000 1.383848000 -1.428224000

C -1.680835000 1.632243000 -1.671140000

C -2.067383000 2.565382000 -2.618113000

C -1.087783000 3.255499000 -3.329730000

C 0.251528000 2.993009000 -3.075673000

C 0.586204000 2.047961000 -2.115329000

N -1.938025000 -0.000749000 -0.000304000

C -2.591259000 0.831559000 -0.851352000

C -3.974217000 0.852136000 -0.872303000

C -4.661712000 0.001279000 -0.000701000

C -3.975743000 -0.850309000 0.871467000

C -2.592799000 -0.831618000 0.850951000

N -0.359051000 -1.387201000 1.427655000

C -1.683339000 -1.634410000 1.670076000

C -2.070851000 -2.567968000 2.616128000

C -1.091887000 -3.259594000 3.327277000

C 0.247750000 -2.998695000 3.073082000

C 0.583520000 -2.053223000 2.113482000

N 0.355874000 1.430456000 1.382493000

C 1.679600000 1.677613000 1.627866000

C 2.065147000 2.629244000 2.556613000

C 1.084676000 3.342278000 3.244096000

C -0.254314000 3.084339000 2.984248000

C -0.588174000 2.118597000 2.044398000

N 1.937919000 -0.000086000 0.001349000

C 2.590703000 0.856374000 0.829158000

C 3.973619000 0.879330000 0.848922000

C 4.661679000 0.002728000 0.003639000

C 3.976456000 -0.874798000 -0.842892000

C 2.593422000 -0.854404000 -0.826043000

N 0.360190000 -1.430770000 -1.383884000

C 1.684726000 -1.676232000 -1.626713000

C 2.073198000 -2.626155000 -2.556084000

C 1.095056000 -3.339740000 -3.246206000

C -0.244833000 -3.084335000 -2.988002000

C -0.581609000 -2.119847000 -2.047922000

H -3.115685000 2.753745000 -2.801829000

H -1.374901000 3.987600000 -4.072916000

H 1.042341000 3.504875000 -3.605909000

H 1.619516000 1.822918000 -1.899331000

H -4.515000000 1.505812000 -1.540078000

H -4.517673000 -1.502353000 1.539912000

H -3.119347000 -2.756354000 2.798674000

H -1.379748000 -3.991643000 4.070230000

H 1.038033000 -3.512179000 3.602574000

H 1.617218000 -1.829159000 1.897898000

H 3.113217000 2.814590000 2.744770000

H 1.370946000 4.089404000 3.972518000

H -1.045599000 3.615339000 3.494613000

H -1.621318000 1.898480000 1.822505000

H 4.513780000 1.552896000 1.497092000

H 4.519479000 -1.546572000 -1.490579000

H 3.121825000 -2.810747000 -2.741658000

H 1.383772000 -4.085660000 -3.974897000

H -1.034519000 -3.616223000 -3.499908000

H -1.615586000 -1.901698000 -1.827638000

Cl -6.377088000 0.002113000 -0.001758000

Cl 6.377034000 0.003900000 0.004722000

# [Mn(4'-OH-tpy)_2_]^4+^, *S = 3/2*

61

symmetry cs

Mn -0.009602000 0.004451000 0.000000000

N -0.006770000 -0.349995000 1.992390000

N -0.006770000 -0.349995000 -1.992390000

N -0.024933000 -1.931804000 0.000000000

N 0.015216000 1.925734000 0.000000000

N 1.986663000 0.327153000 0.000000000

N -1.999391000 0.381744000 0.000000000

C 0.009436000 0.591480000 2.950764000

H 0.017168000 1.625933000 2.640597000

C -0.015977000 -1.674003000 2.336140000

C -0.015977000 -1.674003000 -2.336140000

C 0.009436000 0.591480000 -2.950764000

H 0.017168000 1.625933000 -2.640597000

C 0.016384000 0.253016000 -4.296776000

C 0.005726000 -1.087780000 -4.658433000

C -0.011392000 -2.063826000 -3.664603000

C 0.016384000 0.253016000 4.296776000

C -0.011392000 -2.063826000 3.664603000

C 0.005726000 -1.087780000 4.658433000

H 0.030061000 1.041367000 -5.036392000

H 0.010737000 -1.376857000 -5.701177000

H 0.010737000 -1.376857000 5.701177000

H 0.030061000 1.041367000 5.036392000

H -0.018883000 -3.113208000 3.922074000

H -0.018883000 -3.113208000 -3.922074000

C -0.027484000 -2.586106000 1.189897000

C -0.027484000 -2.586106000 -1.189897000

C -0.035676000 -3.967960000 -1.216812000

C -0.036999000 -4.666112000 0.000000000

C -0.035676000 -3.967960000 1.216812000

H -0.044747000 -4.520795000 2.145432000

H -0.044747000 -4.520795000 -2.145432000

C 2.929914000 -0.630257000 0.000000000

H 2.603380000 -1.659580000 0.000000000

C 2.350282000 1.645208000 0.000000000

C -2.325429000 1.709797000 0.000000000

C -2.970872000 -0.546624000 0.000000000

H -2.676697000 -1.585631000 0.000000000

C -4.312046000 -0.190157000 0.000000000

C -4.655660000 1.155443000 0.000000000

C -3.648420000 2.117521000 0.000000000

C 4.280492000 -0.313132000 0.000000000

C 3.684882000 2.013939000 0.000000000

C 4.662740000 1.022333000 0.000000000

H -5.062052000 -0.968753000 0.000000000

H -5.694352000 1.458749000 0.000000000

H 5.709940000 1.294894000 0.000000000

H 5.007947000 -1.112815000 0.000000000

H 3.959200000 3.059024000 0.000000000

H -3.892878000 3.170169000 0.000000000

C 1.215667000 2.572600000 0.000000000

C -1.164619000 2.604458000 0.000000000

C -1.179153000 3.980669000 0.000000000

C 0.052287000 4.668878000 0.000000000

C 1.264390000 3.945106000 0.000000000

H 2.199764000 4.486212000 0.000000000

H -2.114415000 4.523635000 0.000000000

O 0.152563000 5.982487000 0.000000000

H -0.708950000 6.426632000 0.000000000

O -0.121581000 -6.009552000 0.000000000

H 0.747594000 -6.435925000 0.000000000

# [Mn(4'-pyrr-tpy)_2_]^4+^, *S = 3/2*

83

symmetry c1

Mn -0.000018000 -0.000045000 -0.000047000

N 0.349600000 -1.412610000 -1.406807000

N 1.903625000 0.000022000 0.000005000

N 0.349453000 1.412560000 1.406722000

N 6.020319000 0.000120000 0.000127000

N -0.349541000 -1.411079000 1.408155000

N -1.903662000 -0.000036000 -0.000028000

N -0.349554000 1.410910000 -1.408329000

N -6.020351000 0.000056000 0.000090000

C -0.594244000 -2.086759000 -2.085231000

H -1.625295000 -1.852520000 -1.865311000

C -0.264308000 -3.044539000 -3.031408000

H -1.054030000 -3.564391000 -3.555648000

C 1.076962000 -3.311456000 -3.281350000

H 1.363187000 -4.054292000 -4.014440000

C 2.055192000 -2.613887000 -2.580049000

H 3.102796000 -2.807498000 -2.761395000

C 1.670325000 -1.664993000 -1.644094000

C 2.582407000 -0.849208000 -0.831361000

C 3.948718000 -0.878312000 -0.853746000

H 4.462889000 -1.549135000 -1.523129000

C 4.693070000 0.000102000 0.000078000

C 3.948624000 0.878487000 0.853850000

H 4.462720000 1.549344000 1.523258000

C 2.582317000 0.849302000 0.831398000

C 1.670148000 1.665035000 1.644078000

C 2.054909000 2.613969000 2.580036000

H 3.102493000 2.807642000 2.761436000

C 1.076599000 3.311500000 3.281263000

H 1.362738000 4.054368000 4.014354000

C -0.264640000 3.044504000 3.031241000

H -1.054424000 3.564328000 3.555416000

C -0.594466000 2.086680000 2.085070000

H -1.625491000 1.852388000 1.865093000

C 6.858459000 -0.928317000 -0.798955000

H 6.807407000 -0.636238000 -1.852261000

H 6.491832000 -1.949737000 -0.696376000

C 8.263811000 -0.732974000 -0.225285000

H 8.412174000 -1.395857000 0.630540000

H 9.031951000 -0.953551000 -0.965641000

C 8.263785000 0.733234000 0.225750000

H 8.412228000 1.396117000 -0.630060000

H 9.031851000 0.953815000 0.966181000

C 6.858375000 0.928570000 0.799282000

H 6.807225000 0.636499000 1.852585000

H 6.491754000 1.949987000 0.696661000

C 0.594383000 -2.084450000 2.087239000

H 1.625405000 -1.850250000 1.867147000

C 0.264561000 -3.041406000 3.034290000

H 1.054346000 -3.560664000 3.559023000

C -1.076676000 -3.308303000 3.284435000

H -1.362810000 -4.050531000 4.018176000

C -2.054991000 -2.611497000 2.582494000

H -3.102573000 -2.805089000 2.763992000

C -1.670234000 -1.663398000 1.645689000

C -2.582392000 -0.848449000 0.832212000

C -3.948700000 -0.877536000 0.854705000

H -4.462831000 -1.547712000 1.524768000

C -4.693102000 0.000044000 0.000065000

C -3.948707000 0.877599000 -0.854608000

H -4.462841000 1.547806000 -1.524636000

C -2.582398000 0.848431000 -0.832207000

C -1.670248000 1.663314000 -1.645761000

C -2.055014000 2.611403000 -2.582574000

H -3.102596000 2.805081000 -2.763978000

C -1.076705000 3.308077000 -3.284656000

H -1.362845000 4.050294000 -4.018405000

C 0.264533000 3.041050000 -3.034655000

H 1.054312000 3.560183000 -3.559522000

C 0.594363000 2.084125000 -2.087575000

H 1.625386000 1.849815000 -1.867602000

C -6.858438000 -0.927627000 0.800104000

H -6.807330000 -0.634546000 1.853129000

H -6.491806000 -1.949140000 0.698475000

C -8.263826000 -0.732838000 0.226333000

H -9.031920000 -0.952714000 0.966944000

H -8.412235000 -1.396537000 -0.628850000

C -8.263833000 0.732939000 -0.226103000

H -9.031946000 0.952812000 -0.966697000

H -8.412226000 1.396637000 0.629083000

C -6.858459000 0.927733000 -0.799906000

H -6.807374000 0.634650000 -1.852932000

H -6.491834000 1.949250000 -0.698289000

# [Mn(4-Ph-tpy)_2_]^4+^, *S = 3/2*

79

symmetry c1

Mn 0.000001000 0.004074000 0.004561000

N 0.348726000 -1.399544000 -1.408738000

N 1.925256000 -0.001038000 -0.000473000

C -0.597988000 -2.065158000 -2.090810000

H -1.629509000 -1.830216000 -1.875013000

C -0.269350000 -3.020295000 -3.041612000

H -1.061910000 -3.533167000 -3.568302000

C 1.069781000 -3.292362000 -3.291626000

H 1.353275000 -4.033211000 -4.027604000

C 2.051873000 -2.601571000 -2.586267000

H 3.099059000 -2.797872000 -2.767363000

C 1.670869000 -1.656142000 -1.647437000

C 2.584899000 -0.849816000 -0.833697000

C 3.963415000 -0.870393000 -0.851097000

H 4.477744000 -1.528145000 -1.535131000

C 4.697269000 -0.006987000 -0.007417000

C 6.161022000 -0.010106000 -0.011196000

C 6.874970000 -1.171878000 -0.368020000

H 6.348148000 -2.085494000 -0.612197000

C 8.261862000 -1.174125000 -0.361301000

H 8.798764000 -2.078468000 -0.619105000

C 8.962297000 -0.016081000 -0.018539000

H 10.045668000 -0.018436000 -0.021307000

N -0.354500000 1.420747000 -1.393443000

N 0.361545000 1.404091000 1.416868000

N -0.355700000 -1.405331000 1.409501000

N -1.925222000 0.003516000 0.003174000

C 0.589615000 2.102542000 -2.063057000

C -0.579213000 2.072588000 2.104354000

C 0.587905000 -2.073965000 2.092919000

H 1.622058000 1.880661000 -1.837830000

H -1.612702000 1.841450000 1.893501000

H 1.620483000 -1.842712000 1.877953000

C 0.256898000 3.058706000 -3.011377000

C -0.241548000 3.025677000 3.054036000

C 0.254473000 -3.027533000 3.043608000

H 1.047228000 3.584554000 -3.528543000

H -1.029045000 3.541341000 3.585587000

H 1.044357000 -3.542861000 3.571926000

C -1.083473000 3.314818000 -3.271555000

C 1.100073000 3.292333000 3.296655000

C -1.086034000 -3.294653000 3.291741000

H -1.370152000 4.054922000 -4.007049000

H 1.390557000 4.031612000 4.031490000

H -1.373262000 -4.033676000 4.028110000

C -2.062705000 2.610181000 -2.575984000

C 2.075684000 2.598258000 2.585556000

C -2.064707000 -2.601216000 2.584236000

H -3.110709000 2.795944000 -2.763290000

H 3.124537000 2.790450000 2.761128000

H -3.112796000 -2.793823000 2.763945000

C -1.677753000 1.665421000 -1.638088000

C 1.685964000 1.654813000 1.648288000

C -1.679078000 -1.657324000 1.645728000

C -2.588440000 0.850775000 -0.828618000

C 2.592579000 0.844720000 0.829447000

C -2.589081000 -0.847557000 0.830576000

C 3.971305000 0.859476000 0.840062000

C -3.967683000 -0.867595000 0.841598000

C -3.967039000 0.865018000 -0.846673000

H 4.491950000 1.514892000 1.521599000

H -4.485395000 -1.526539000 1.521930000

H -4.483980000 1.521771000 -1.529696000

C -4.697396000 -0.002559000 -0.004130000

C -6.161142000 -0.004906000 -0.007051000

C 6.881779000 1.148607000 0.341855000

C -6.877487000 -1.165846000 0.347799000

C -6.879733000 1.153884000 -0.364431000

H 6.360141000 2.064487000 0.588641000

H -6.352463000 -2.079576000 0.595331000

H -6.356641000 2.069333000 -0.609709000

C 8.268618000 1.144936000 0.327853000

C -8.264323000 -1.167235000 0.335029000

C -8.266609000 1.151002000 -0.356819000

H 8.810734000 2.047005000 0.582689000

H -8.802905000 -2.070924000 0.591607000

H -8.807007000 2.053091000 -0.615213000

C -8.962547000 -0.009193000 -0.012216000

H -10.045920000 -0.010900000 -0.014281000

# [Mn(4'-(4-MePh)-tpy)_2_]^4+^, *S = 3/2*

85

symmetry c1

Mn -0.000115000 0.004775000 -0.005951000

N -0.348050000 -1.397416000 1.409148000

N -1.920921000 -0.000305000 -0.002311000

C 0.597807000 -2.062590000 2.092935000

H 1.629288000 -1.827516000 1.876771000

C 0.268873000 -3.016547000 3.044521000

H 1.060748000 -3.529120000 3.572559000

C -1.070711000 -3.288261000 3.293727000

H -1.354827000 -4.028346000 4.030269000

C -2.051886000 -2.598325000 2.586898000

H -3.099141000 -2.794665000 2.767369000

C -1.669943000 -1.653610000 1.647128000

C -2.583523000 -0.847874000 0.831265000

C -3.960741000 -0.869333000 0.848067000

H -4.473071000 -1.526515000 1.533874000

C -4.698092000 -0.006622000 0.002893000

C -6.156089000 -0.010095000 0.006249000

C -6.878078000 -1.152154000 0.412897000

H -6.357740000 -2.058199000 0.695923000

C -8.261572000 -1.154269000 0.403272000

H -8.793560000 -2.051512000 0.696960000

C -8.986883000 -0.018096000 0.011733000

C -10.488346000 -0.009778000 0.040840000

H -10.843447000 0.433958000 0.977927000

H -10.895235000 -1.020014000 -0.020368000

H -10.897995000 0.588502000 -0.775396000

N 0.351832000 1.423756000 1.391006000

N -0.359496000 1.402695000 -1.421484000

N 0.355742000 -1.406536000 -1.409466000

N 1.920744000 0.004675000 -0.002744000

C -0.592561000 2.105663000 2.060162000

C 0.580914000 2.069737000 -2.110855000

C -0.586598000 -2.076888000 -2.093004000

H -1.624598000 1.882370000 1.834062000

H 1.614158000 1.837308000 -1.900027000

H -1.619302000 -1.845567000 -1.878291000

C -0.261223000 3.062537000 3.007972000

C 0.244006000 3.021647000 -3.061740000

C -0.252336000 -3.031150000 -3.042431000

H -1.051827000 3.588471000 3.524654000

H 1.031455000 3.536145000 -3.594515000

H -1.041287000 -3.547819000 -3.570859000

C 1.079142000 3.319445000 3.268537000

C -1.097729000 3.288820000 -3.304184000

C 1.088761000 -3.297561000 -3.289327000

H 1.365146000 4.060181000 4.003692000

H -1.388065000 4.027234000 -4.039980000

H 1.377046000 -4.037054000 -4.024843000

C 2.058703000 2.614742000 2.574090000

C -2.073136000 2.596228000 -2.591938000

C 2.066134000 -2.602932000 -2.581790000

H 3.106465000 2.801104000 2.762020000

H -3.121888000 2.788717000 -2.767685000

H 3.114407000 -2.795266000 -2.760561000

C 1.674431000 1.668857000 1.636470000

C -1.683463000 1.653635000 -1.653242000

C 1.679049000 -1.658107000 -1.644265000

C 2.585850000 0.852893000 0.828184000

C -2.590408000 0.844249000 -0.833212000

C 2.588045000 -0.847023000 -0.828314000

C -3.967851000 0.859576000 -0.844913000

C 3.965291000 -0.867958000 -0.837979000

C 3.963151000 0.867814000 0.847870000

H -4.485687000 1.514312000 -1.528924000

H 4.481115000 -1.527927000 -1.518441000

H 4.476728000 1.526135000 1.531626000

C 4.698272000 -0.001920000 0.007783000

C 6.156222000 -0.005288000 0.012016000

C -6.886357000 1.127720000 -0.400174000

C 6.880620000 -1.146825000 -0.392020000

C 6.884560000 1.132232000 0.423062000

H -6.371936000 2.034749000 -0.690687000

H 6.362195000 -2.052802000 -0.678595000

H 6.368900000 2.039041000 0.711842000

C -8.268940000 1.121539000 -0.387035000

C 8.264062000 -1.148680000 -0.375833000

C 8.267090000 1.126178000 0.417118000

H -8.807229000 2.013092000 -0.687184000

H 8.797593000 -2.045433000 -0.668172000

H 8.803731000 2.017418000 0.721131000

C 8.987265000 -0.012896000 0.020524000

C 10.488804000 -0.003849000 -0.000835000

H 10.893803000 0.582876000 0.826127000

H 10.895909000 -1.014611000 0.048019000

H 10.848562000 0.454019000 -0.929288000
